# Opium use and subsequent incidence of cancer: results from the Golestan Cohort Study

**DOI:** 10.1016/S2214-109X(20)30059-0

**Published:** 2020-04-27

**Authors:** Mahdi Sheikh, Ramin Shakeri, Hossein Poustchi, Akram Pourshams, Arash Etemadi, Farhad Islami, Masoud Khoshnia, Abdolsamad Gharavi, Gholamreza Roshandel, Hooman Khademi, Sadaf G Sepanlou, Maryam Hashemian, Abdolreza Fazel, Mahdi Zahedi, Behnoush Abedi-Ardekani, Paolo Boffetta, Sanford M Dawsey, Paul D Pharoah, Masoud Sotoudeh, Neal D Freedman, Christian C Abnet, Nicholas E Day, Paul Brennan, Farin Kamangar, Reza Malekzadeh

**Affiliations:** a Digestive Oncology Research Center, Digestive Diseases Research Institute, Tehran University of Medical Sciences, Tehran, Iran; b Liver and Pancreatobiliary Diseases Research Center, Digestive Diseases Research Institute, Tehran University of Medical Sciences, Tehran, Iran; c Digestive Disease Research Center, Digestive Diseases Research Institute, Tehran University of Medical Sciences, Tehran, Iran; d Section of Genetics, International Agency for Research on Cancer, WHO, Lyon, France; e Metabolic Epidemiology Branch, Division of Cancer Epidemiology and Genetics, National Cancer Institute, Bethesda, MD, USA; f Surveillance and Health Services Research, American Cancer Society, Atlanta, GA, USA; g Golestan Research Center of Gastroenterology and Hepatology, Golestan University of Medical Sciences, Gorgan, Iran; h Cancer Research Center, Golestan University of Medical Sciences, Gorgan, Iran; i Ischemic Disorders Research Center, Golestan University of Medical Sciences, Gorgan, Iran; j Department of Biology, School of Arts and Sciences, Utica College, Utica, NY, USA; k Tisch Cancer Institute, Icahn School of Medicine at Mount Sinai, New York, NY, USA; l Department of Medical and Surgical Sciences, University of Bologna, Bologna, Italy; m Department of Public Health and Primary Care, University of Cambridge, Cambridge, UK; n Department of Biology, School of Computer, Mathematical, and Natural Sciences, Morgan State University, Baltimore, MD, USA

## Abstract

**Background:**

Evidence is emerging for a role of opiates in various cancers. In this study, we aimed to investigate the association between regular opium use and cancer incidence.

**Methods:**

This study was done in a population-based cohort of 50 045 individuals aged 40–75 years from northeast Iran. Data on participant demographics, diet, lifestyle, opium use, and different exposures were collected upon enrolment using validated questionnaires. We used proportional hazards regression models to estimate hazard ratios (HRs) and corresponding 95% CIs for the association between opium use and different cancer types.

**Findings:**

During a median 10 years of follow-up, 1833 participants were diagnosed with cancer. Use of opium was associated with an increased risk of developing all cancers combined (HR 1·40, 95% CI 1·24–1·58), gastrointestinal cancers (1·31, 1·11–1·55), and respiratory cancers (2·28, 1·58–3·30) in a dose-dependent manner (p_trend_<0·001). For site-specific cancers, use of opium was associated with an increased risk of developing oesophageal (1·38, 1·06–1·80), gastric (1·36, 1·03–1·79), lung (2·21, 1·44–3·39), bladder (2·86, 1·47–5·55), and laryngeal (2·53, 1·21–5·29) cancers in a dose-dependent manner (p_trend_<0·05). Only high-dose opium use was associated with pancreatic cancer (2·66, 1·23–5·74). Ingestion of opium (but not smoking opium) was associated with brain (2·15, 1·00–4·63) and liver (2·46, 1·23–4·95) cancers in a dose-dependent manner (p_rend_<0·01). We observed consistent associations among ever and never tobacco users, men and women, and individuals with lower and higher socioeconomic status.

**Interpretation:**

Opium users have a significantly higher risk of developing cancers in different organs of the respiratory, digestive, and urinary systems and the CNS. The results of this analysis show that regular use of opiates might increase the risk of a range of cancer types.

**Funding:**

World Cancer Research Fund International, Cancer Research UK, Tehran University of Medical Sciences, US National Cancer Institute, International Agency for Research on Cancer.

## Introduction

The opiate crisis has resulted in thousands of deaths annually and billions in economic losses in many parts of the world.^1^ In 2017, an estimated 29·2 million people used opiates, mainly as illicit drugs.^1^ In addition to the acute health hazards of opiate misuse, reports on the association between use of opium (the raw extract of opium poppy) and some cancers have raised concerns about the long-term effects of using opiates.^2^

Previous studies have linked opium use with oesophageal,^3^ gastric,^4^, ^5^ pancreatic,^6^ laryngeal,^7^, ^8^ lung,^9^, ^10^ and bladder^11^ cancers. Most of these studies have a case-control design with substantial limitations and potential biases. Opium and its derivatives are widely used for pain management, including cancer pain, and thus reverse causality is a major concern in these studies.^2^ Many opium users are also heavy tobacco smokers; therefore confounding by tobacco use and other socioeconomic factors might be present and incompletely adjusted for in previous studies. Furthermore, most published studies have not assessed different types, routes, and doses of opium, which differ in their bioavailabilities, carcinogenic metabolites, and by-products.^2^, ^12^, ^13^ There is evidence from experimental studies that opiates might play a part in other cancer types including liver^14^, ^15^ and brain^16^, ^17^ cancers, although epidemiological studies of these possible associations are needed.

Because of the challenges inherent in studying opium, including gathering valid consumption data and the fear of stigma and prosecution, prospective studies of opium use with long-term follow-up are lacking. The Golestan Cohort Study (GCS) is the only population-based prospective study that includes a large group of regular opium users with validated opium use data.^18^, ^19^ After a median of 10 years' follow-up, we provide the first report from the GCS on the association between regular opium use and overall and site-specific cancer incidence, and further examine these associations among different routes, doses, and types of opium.

**Research in context**

**Evidence before this study**

The global crisis of opiate misuse and emerging evidence from experimental and case-control studies showing possible carcinogenic effects of some opium derivatives have raised substantial concern over the long-term effects of using opiates. We searched PubMed, Web of Science, and Scopus on Nov 1, 2019, for publications about use of opiates and cancer risk using the following Medical Subject Headings and relevant terms (opium OR opiate* OR opioid*) AND (neoplasm* OR carcinogen* OR malignan* OR tumor* tumour* OR cancer*), with no language or date restrictions. Because of the challenges inherent in studying exposure, there is a paucity of prospective studies of opium use with long-term follow-up. Opium and its derivatives are widely used for cancer pain management and thus reverse causality is a major concern in the available case-control studies. Furthermore, most published studies identified by our search did not assess different types, routes, and doses of opium, which differ in their bioavailabilities, carcinogenic metabolites, and by-products. Additionally, some associations between opiates and specific cancer types have only been described in experimental studies and epidemiological investigations of these associations are lacking.

**Added value of this study**

To our knowledge, the Golestan Cohort Study (GCS) is the only population-based prospective study that includes a large group of regular opium users with validated opium use data. Furthermore, this report is the only prospective analysis from human studies on associations between opium use and overall and site-specific cancer incidence and is based on more than a decade of following up 50 000 participants of the GCS. This study documents the presence of dose-dependent associations between opium use and a broad array of cancer types, including oesophageal, gastric, laryngeal, lung, pancreatic, liver, bladder, and brain. We observed consistent associations between opium use and cancer among ever and never tobacco users, men and women, and individuals with lower and higher socioeconomic status. Furthermore, all routes and types of opium derivatives used by this population showed evidence of carcinogenic effects. Therefore, this study has important implications for public health and could aid the translation of knowledge and implementation of evidence into practice and policy decision making.

**Implications of all the available evidence**

The overall evidence indicates that the carcinogenic effects of regular use of opium derivatives might be greater than expected and extend beyond the upper aerodigestive tract to include several organs of the respiratory, digestive, and urinary systems and the CNS. Given the huge increase in use of opiates in the past few years, further global initiatives to reduce opiate misuse and implement preventive strategies to mitigate their hazardous long-term effects are needed.

## Methods

### Study design and population

The design and objectives of the GCS have been published previously.^18^ Briefly, the main purpose of the GCS was to study risk factors for chronic diseases, with an emphasis on oesophageal cancer because of its high incidence in the study area. After completion of a pilot study, between January, 2004, and June, 2008, 50 045 individuals, aged 40–75 years and from rural and urban areas of the Golestan province in northeast Iran, were enrolled into the study. Individuals who had been diagnosed with upper gastrointestinal cancers before enrolment, those who were unwilling to participate, and temporary residents were excluded. The urban participants were selected randomly by systemic clustering, using household numbers, and were then contacted and invited by trained staff to participate in the study. In rural areas, all eligible people living in the 326 villages of the study area were contacted and invited to participate. This process was done using the primary health-care networks present in each group of villages and usually staffed by two local health-care workers. All participants provided written informed consent before enrolment.

The GCS was approved by the institutional review boards of the Digestive Disease Research Institute of Tehran University of Medical Sciences, the International Agency for Research on Cancer, and the US National Cancer Institute.

### Questionnaires and data gathering

Upon enrolment, two validated questionnaires were completed for the participants: a detailed general questionnaire (collecting data on demographics, socioeconomic status, lifestyle, and different exposures)^18^ and a food frequency questionnaire (FFQ) that included 116 food items with portion size photos and questions about the frequency and amount of consumption of each item. Details of interviews and validation studies are described in the appendix (p 1). The general questionnaire included questions about consumption of opium, cigarettes, alcohol, hookah, and nass (a chewing tobacco product), and the starting and ending ages, frequency, and consumption amount of each agent. We calculated the cumulative smoked cigarettes in pack-years (a pack includes 20 cigarettes), and the cumulative chewed nass in nass-years by calculating the number of units used per day multiplied by the number of consumption years. Because of the small numbers of participants who consumed alcohol and hookah, for these two exposures we categorised participants as ever or never users. Fuel sources for household heating and cooking and the duration of their use were also assessed and participants were categorised based on the household fuel they used in the past 20 years as predominantly using gas, kerosene, biomass, or mixed fuels. To evaluate socioeconomic status, we used the quartiles of a wealth score that was previously created using multiple correspondence analysis of property ownership, structure, and size, vehicle ownership, and possession of certain home appliances. We assessed diet based on the Healthy Eating Index (HEI) that was previously created using FFQ data. The HEI ranges from 0 to 100 and shows general dietary patterns. The HEI was previously created based on daily intake of fruits, vegetables, whole grains, dairy products, proteins, fatty acids, sodium, and added sugars. Details and methods of creating the wealth score and the HEI dietary score are described in the appendix (pp 1–2).

### Definitions and assessment of the main exposure

Opiates are a subgroup of opioids that contain various products derived from the opium poppy plant, including opium, morphine, and heroin.^1^ The main opiate types used in the Golestan region are raw opium (teriak), refined opium (shireh), opium dross (sukhteh), and heroin. Raw opium is the air-dried extract of the opium poppy plant that is acquired through ripening the poppy capsules. Raw opium can be ingested or smoked after direct heating with special devices.^12^ Opium dross is the remnants of smoked opium and can only be ingested. Refined opium is obtained from boiling the opium dross (with or without adding raw opium) in water, filtering the mixture several times, and then evaporating the filtrate.^12^ Refined opium can be ingested or smoked by indirect heating using special devices. Heroin is typically injected into a vein, but can also be smoked.

Literature from the 1980s suggested opium as a potential risk factor for oesophageal cancer.^13^ Therefore, GCS questionnaires included detailed queries about opium use, including the type of opium, route of use, age of starting and stopping use of each opium type through each route, frequency of use, and amount of use in nokhods (local unit, about 0·2 g). We did a validation study of self-reported opium use during the cohort's pilot phase and found high correlation between questionnaire responses and urinary levels of opium metabolites, with a sensitivity and specificity of over 90% for current use.^19^ For this analysis, we defined regular opium users as those who used opium at least once a week for at least 6 months.^12^

### Follow-up and outcome ascertainment

The current analysis is based on 531 789 person-years of follow-up. Participants were followed up from enrolment by annual telephone surveys and home visits. If participants or their families reported incident cancers or deaths, a staff member was sent to the home of the patient or the deceased individual to collect detailed information and a team was sent to the corresponding medical centres to gather copies of relevant medical reports. Collected documents were reviewed separately by two expert physicians to verify the diagnosis of cancer. In cases of disagreement, a third expert physician finalised the diagnosis. The final diagnosis of cancer was recorded based on the Ninth Revision of the International Statistical Classification of Diseases and Related Health Problems. For this study, we matched the recorded cancer cases to the Golestan Population-based Cancer Registry database to minimise any possible misclassifications. 1833 (92%) of 1991 self-reported cancer cases were confirmed using the explained quality control steps and were included in this analysis. We used first primary cancer cases for our site-specific analysis. For gastric cancer, we did a subgroup analysis of cardia and non-cardia subtypes. Most oesophageal cancer cases were squamous cell carcinoma so we did not do a subgroup analysis by histology.

### Statistical analysis

We used Cox proportional hazards regression models to estimate hazard ratios (HRs) and corresponding 95% CIs for the association between opium use and risk of cancer. We set age as the timescale and defined the entry time as the age at enrolment in the GCS. The exit time was defined as the age at first cancer diagnosis for the cancer cases, the age at death for deaths from other causes, and the age at last follow-up for other participants, until Jan 1, 2019 (exit date was censored at this point).

We used two models for this analysis. The first model (model 1) included sex (male *vs* female), ethnicity (Turkman *vs* non-Turkman), residence (urban *vs* rural), wealth score (quartiles), smoking cigarettes (ever *vs* never), cumulative pack-years of smoked cigarettes (continuous), and regular alcohol drinking (never *vs* ever). The second model (model 2) additionally included chewing nass (never *vs* two quantiles of the cumulative nass-years), regular consumption of hookah (never *vs* ever), predominant household fuel (natural gas *vs* kerosene *vs* biomass *vs* mixed), and diet (tertiles of the HEI score). The results of the two models were very similar (appendix p 6); therefore, we used the more parsimonious model for subsequent analyses. Before using this model for each cancer outcome, we tested the proportional hazards assumption using Schoenfeld's global test. None of the opium use variables violated the proportional hazards assumption; however, some covariates showed evidence of time-varying effects in certain models and therefore were treated as time-varying covariates, allowing for time-by-covariate interaction within the corresponding model.

We examined ever-use of opium and assessed if the effect of opium use varied by whether it was smoked or ingested, since previous evidence suggested that consumption patterns might affect compound exposure.^2^, ^12^, ^13^ We calculated the cumulative use of opium via any route, as well as separately for opium smoking and opium ingestion by calculating the number of nokhods (0·2 g) used per day through that specific route multiplied by the number of consumption-years. Only 520 (6·1%) of 8486 opium users consumed opium through both routes. For these dual-route users, we calculated the cumulative amount of ingested and smoked opium separately and included them in the corresponding categories of both opium ingestion and opium smoking.

For assessing the dose–response relationships, we categorised the cumulative opium used into quartiles of nokhod-years. To calculate the p value for trends we used two methods: first, we assigned consecutive integers to these consecutive categories, and then we assigned the median values for each category. Both methods provided similar results; therefore only the results of the first method are presented in this paper.

In addition to adjusting for two tobacco-related variables (indicating the status and intensity of tobacco use) in the main model, we stratified the analyses by ever versus never use of tobacco and further assessed the interaction between opium and tobacco use because of concerns about residual confounding from tobacco smoking. The questionnaire responses on tobacco use were validated previously through reinterviewing a subgroup of participants and comparing questionnaire responses with the presence of cotinine in their urine samples.^18^ Sensitivity analyses were done by repeating the analyses after exclusion of the first 2 years of follow-up, by excluding cancer cases without histological confirmation, by stratifying the analyses by socioeconomic status and sex, and by using the interaction test to assess effect modification. All statistical analyses were two-sided and done using Stata statistical software version 14.

### Role of the funding source

The funders of the study had no role in study design, data collection, data analysis, data interpretation, or writing of the report. The corresponding authors had full access to all the data in the study and had final responsibility for the decision to submit for publication.

## Results

50 045 participants were enrolled in the GCS. We excluded 11 individuals who had been diagnosed with cancer before enrolment, leaving 50 034 participants for this analysis. During follow-up, 1833 (3·7%) participants were diagnosed with various cancers—1464 (79·9%) of these had histological confirmation and 369 (20·1%) were identified through verbal autopsy and other available medical records. Participants who developed cancer tended to be older, male, Turkman, and have a lower wealth score (table 1). Furthermore, cancer incidence was higher among participants who consumed an unhealthy diet, burned biomass as the main household fuel, smoked cigarettes, chewed nass, and consumed alcohol (table 1).

**Table 1:** Baseline characteristics of all cohort participants and individuals who developed cancer during the follow-up period

		**Cancer group (n=1833)**	**Non-cancer group (n=48 201)**	**Entire cohort (n=50 034)**	**p value**
Age (years)		57·63 (9·4)	51·8 (8·8)	52·05 (8·9)	<0·0001
Sex		..	..	..	<0·0001
	Male	1002 (54·6%)	20 226 (42·0%)	21 228 (42·4%)	..
	Female	831 (45·3%)	27 975 (58·0%)	28 806 (57·6%)	..
Ethnicity		..	..	..	0·0041
	Turkman	1417 (77·3%)	35 828 (74·3%)	37 245 (74·4%)	..
	Non-Turkman	416 (22·7%)	12 373 (25·7%)	12 789 (25·6%)	..
Residence		..	..	..	0·201
	Rural	1487 (81·1%)	38 515 (79·9%)	40 002 (79·9%)	..
	Urban	346 (18·9%)	9686 (20·1%)	10 032 (20·1%)	..
Wealth score		..	..	..	<0·0001
	First quartile (lowest)	622 (33·9%)	13 310 (27·6%)	13 932 (27·8%)	..
	Second quartile	404 (22·0%)	10 740 (22·3%)	11 144 (22·3%)	..
	Third quartile	440 (24·0%)	12 142 (25·2%)	12 582 (25·1%)	..
	Fourth quartile (highest)	367 (20·0%)	12 009 (24·9%)	12 376 (24·7%)	..
Healthy Eating Index*		..	..	..	0·0045
	Lowest tertile (≤30)	697 (38·9%)	16 986 (35·9%)	17 683 (36·0%)	..
	Middle tertile (31–38)	613 (34·2%)	15 973 (33·7%)	16 586 (33·8%)	..
	Highest tertile (≥39)	482 (26·9%)	14 306 (30·2%)	14 788 (30·1%)	..
Predominant household fuel†		..	..	..	0·045
	Natural gas	211 (11·5%)	5823 (12·1%)	6034 (12·1%)	..
	Mixed fuels	161 (8·8%)	4286 (9·0%)	4447 (8·9%)	..
	Kerosene	1284 (70·0%)	34 187 (71·7%)	35 471 (71·6%)	..
	Biomass	160 (8·7%)	3378 (7·0%)	3538 (7·1%)	..
Smoking (pack-years)		..	..	..	<0·0001
	Never	1361 (74·2%)	40 017 (83·0%)	41 378 (82·7%)	..
	Lowest tertile (<5·7)	114 (6·2%)	2779 (5·8%)	2893 (5·8%)	..
	Middle tertile (5·7–20)	142 (7%)	2851 (9%)	2993 (6·0%)	..
	Highest tertile (>20)	216 (11·8%)	2554 (5·3%)	2770 (5·5%)	..
Nass chewing (nass-years)		..	..	..	<0·0001
	Never	1584 (86·4%)	44 610 (92·5%)	46 194 (92·3%)	..
	Lower than median	117 (6·4%)	1899 (3·9%)	2016 (4·0%)	..
	Higher than median	132 (7·2%)	1692 (3·5%)	1824 (3·6%)	..
Regular alcohol drinking		..	..	..	0·0003
	Never	1743 (95·1%)	46 582 (96·6%)	48 325 (96·6%)	..
	Ever	90 (4·9%)	1619 (3·4%)	1709 (3·4%)	..
Regular hookah use		..	..	..	0·176
	Never	1806 (98·5%)	47 656 (98·9%)	49 462 (98·9%)	..
	Ever	27 (1·5%)	545 (1·1%)	572 (1·1%)	..

Data are mean (SD) or n (%), unless otherwise indicated.

* Details of daily dietary intake were missing for 977 (2%) participants.

† Details of household fuel use were missing for 544 (1·0%) participants.

Details of opium use and the demographics of opium users are shown in table 2. The median duration of opium use was 10·4 years, raw opium was the most commonly used opium type, and smoking was the predominant route of opium use in this population. Opium users tended to be older, male, belong to the Turkman ethnicity, live in rural areas, and have a lower wealth score. Furthermore, consuming an unhealthy diet, burning biomass as the main household fuel, smoking cigarettes, chewing nass, and consuming alcohol were more common among opium users than among never opium users.

**Table 2:** Opium use, and the distribution of baseline characteristics and potential confounders between opium ever versus never users

		**Ever opium users (n=8486)**	**Never opium users (n=41 548)**	**p value**
Duration of opium use (years)		10·4 (4·5–20·2)	..	..
Time of opium use				
	Current	7618 (89·8%)	..	..
	Former	868 (10·2%)	..	..
Type of opium used				
	Raw opium (teriak)	7306 (86·1)	..	..
	Refined opium (shireh)	782 (9·2%)	..	..
	Burned opium (sukhteh)	6 (<0·1%)	..	..
	Heroin	4 (<0·1%)	..	..
	Combination of the above	388 (4·6%)	..	..
Route of opium use				
	Only smoking	5810 (68·5%)	..	..
	Only ingestion	2156 (25·4%)	..	..
	Both routes	520 (6·1%)	..	..
Cumulative amount of opium used (nokhod-years)				
	First quartile (≤5)	2146 (25·2%)	..	..
	Second quartile (5·1–21)	2106 (24·8%)	..	..
	Third quartile (21·1–60)	2124 (25·0%)	..	..
	Fourth quartile (>60)	2110 (24·9%)	..	..
Age (years)		53·27 (9·1)	51·80 (8·8)	<0·0001
Sex		..	..	<0·0001
	Male	6132 (72·3%)	15 096 (36·3%)	..
	Female	2354 (27·7%)	26 452 (63·7%)	..
Ethnicity		..	..	<0·0001
	Turkman	6552 (77·2%)	30 693 (73·9%)	..
	Non-Turkman	1934 (22·8%)	10 855 (26·1%)	..
Residence		..	..	<0·0001
	Rural	7484 (88·2%)	32 518 (78·3%)	..
	Urban	1002 (11·8%)	9030 (21·7%)	..
Wealth score		..	..	<0·0001
	First quartile (lowest)	3274 (38·6%)	10 658 (25·7%)	..
	Second quartile	1955 (23·0%)	9189 (22·1%)	..
	Third quartile	1897 (22·4%)	10 685 (25·7%)	..
	Fourth quartile (highest)	1360 (16·0%)	11 016 (26·5%)	..
Healty eating index*		..	..	<0·0001
	Lowest tertile (≤30)	3524 (42·3%)	14 159 (34·7%)	..
	Middle tertile (31–38)	2736 (32·9%)	13 850 (33·9%)	..
	Highest tertile (≥39)	2054 (24·7%)	12 734 (31·2%)	..
Predominant household fuel†		..	..	<0·0001
	Natural gas	634 (7·5%)	5400 (13·1%)	..
	Mixed fuels	511 (6·0%)	3936 (9·5%)	..
	Kerosene	6537 (77·5%)	28 934 (70·4%)	..
	Biomass	743 (8·8%)	2795 (6·8%)	..
Smoking (pack-years)		..	..	<0·0001
	Never	4011 (47·3%)	37 367 (89·9%)	..
	Lowest tertile (<5·7)	1297 (15·3%)	1596 (3·8%)	..
	Middle tertile (5·7–20)	1551 (18·3%)	1442 (3·5%)	..
	Highest tertile (>20)	1627 (19·2%)	1143 (2·8%)	..
Nass chewing (nass-years)		..	..	<0·0001
	Never	5981 (70·5%)	40 213 (96·8%)	..
	Lower than median	1325 (15·6%)	691 (1·7%)	..
	Higher than median	1180 (13·9%)	644 (1·6%)	..
Regular alcohol drinking		..	..	<0·0001
	Never	7566 (89·2%)	40 759 (98·1%)	..
	Ever	920 (10·8%)	789 (1·9%)	..
Regular hookah use		..	..	<0·0001
	Never	8325 (98·1%)	41 137 (99·0%)	..
	Ever	161 (1·9%)	411 (1·0%)	..

Data are median (IQR), n (%), or mean (SD), unless otherwise indicated.

* Details of daily dietary intake were missing for 977 (1·9%) participants.

† Details of household fuel use were missing for 544 (1·0%) participants.

**Figure 1:**
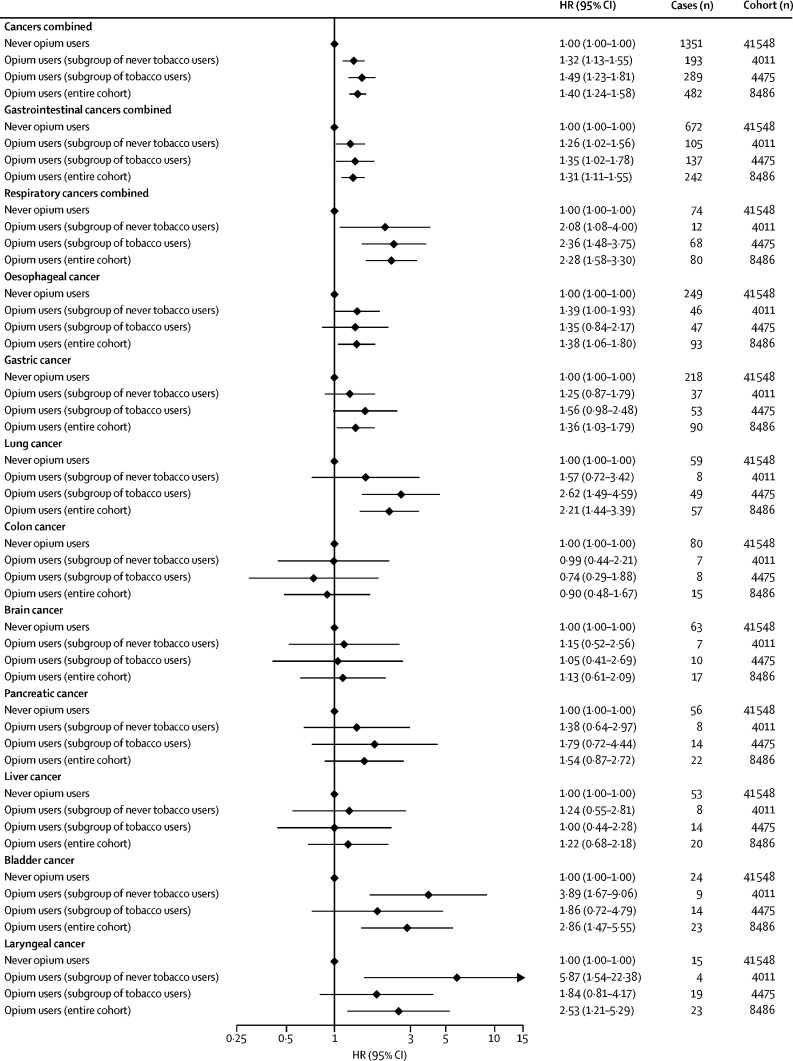
Ever-use of opium and risk of different cancer types among the entire cohort, the tobacco user subgroup, and never tobacco user subgroup This model uses age as the timescale and is adjusted for sex, ethnicity (Turkman *vs* non-Turkman), residence (urban *vs* rural), wealth score quartiles, smoking cigarettes (in the subgroups of tobacco users and entire cohort, fitted as ever *vs* never), cumulative pack-years of smoked cigarettes (in the subgroups of tobacco users and entire cohort, fitted as a continuous variable), and regular alcohol drinking (ever *vs* never). HR=hazard ratio.

**Figure 2:**
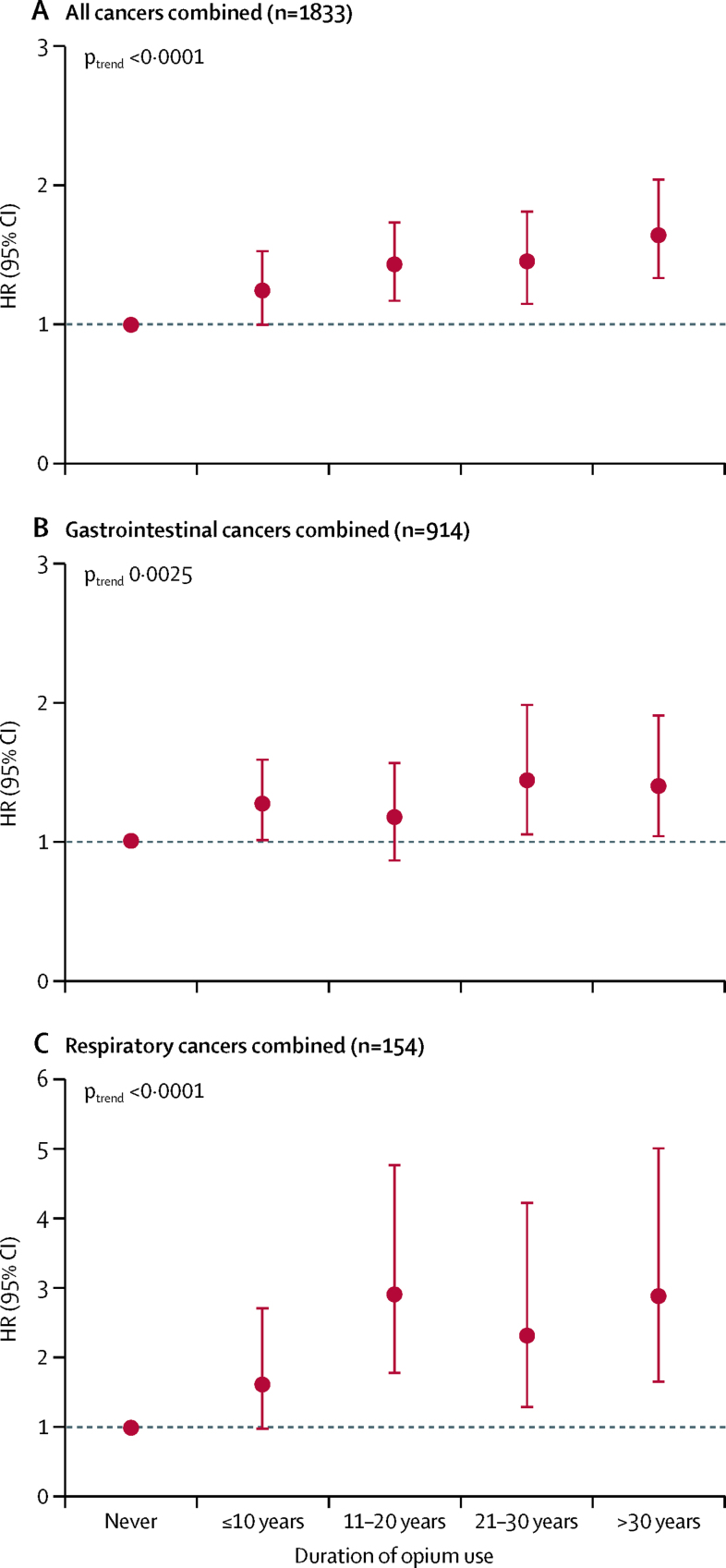
Dose–response associations between duration of opium use and risk of all cancers combined (A), gastrointestinal cancers combined (B), and respiratory cancers combined (C) The y axis shows HRs in a model that used age as the timescale and was adjusted for sex, ethnicity (Turkman *vs* non-Turkman), residence (urban *vs* rural), wealth score quartiles, smoking cigarettes (ever *vs* never), cumulative pack-years of smoked cigarettes (continuous variable), and regular alcohol drinking (never *vs* ever). HR=hazard ratio.

Ever-use of opium and the duration of opium use, regardless of the type and route of use, showed a significant association with the risk of developing all cancers combined, gastrointestinal cancers, and respiratory cancers in the entire cohort, and within never and ever tobacco users (Figure 1, Figure 2; appendix pp 3–5) in a dose-dependent manner (table 3; figure 2; appendix pp 3–5). Although opium use combined with tobacco use seems to have multiplicative effects, particularly on respiratory cancers, the interaction test was not significant, possibly because of the small number of cases (appendix p 7).

**Table 3:** Dose–response associations between different routes of opium use and the risk of developing different cancers

	**Never used this route**	**First quartile**	**Second quartile**	**Third quartile**	**Fourth quartile**	**P_trend_**
**All cancers combined (n=1833)**						
Any route (n=482)	1 (ref)	1·24 (1·01–1·53)	1·22 (0·99–1·51)	1·43 (1·18–1·75)	1·70 (1·42–2·04)	<0·0001
Smoking (n=322)	1 (ref)	1·12 (0·88–1·43)	1·00 (0·77–1·30)	1·33 (1·07–1·67)	1·64 (1·33–2·02)	<0·0001
Ingestion (n=201)	1 (ref)	1·20 (0·88–1·64)	1·53 (1·16–2·02)	1·41 (1·05–1·91)	1·49 (1·14–1·95)	<0·0001
**Gastrointestinal cancers combined (n=914)**						
Any route (n=242)	1 (ref)	1·28 (0·96–1·70)	1·19 (0·89–1·60)	1·28 (0·97–1·70)	1·48 (1·14–1·91)	0·0007
Smoking (n=162)	1 (ref)	1·32 (0·96–1·81)	0·99 (0·68–1·42)	1·22 (0·89–1·69)	1·44 (1·06–1·95)	0·014
Ingestion (n=99)	1 (ref)	1·07 (0·68–1·68)	1·35 (0·90–2·00)	1·33 (0·88–2·01)	1·30 (0·88–1·90)	0·033
**Respiratory cancers combined (n=154)**						
Any route (n=80)	1 (ref)	1·14 (0·54–2·40)	2·38 (1·37–4·11)	2·26 (1·30–3·92)	3·22 (2·02–5·14)	<0·0001
Smoking (n=53)	1 (ref)	0·29 (0·07–1·20)	1·42 (0·73–2·76)	1·76 (0·98–3·14)	2·83 (1·77–4·52)	0·0001
Ingestion (n=36)	1 (ref)	2·16 (1·04–4·48)	2·63 (1·39–4·97)	1·19 (0·48–2·96)	1·94 (1·03–3·68)	0·0081
**Oesophageal cancer (n=342)**						
Any route (n=93)	1 (ref)	1·34 (0·84–2·12)	1·18 (0·73–1·91)	1·42 (0·90–2·21)	1·60 (1·06–2·42)	0·0099
Smoking (n=65)	1 (ref)	1·34 (0·78–2·31)	1·00 (0·54–1·85)	1·62 (1·00–2·61)	1·79 (1·12–2·86)	0·0046
Ingestion (n=37)	1 (ref)	1·34 (0·71–2·54)	1·05 (0·51–2·14)	1·53 (0·83–2·84)	0·91 (0·44–1·87)	0·527
**Gastric cancer (n=308)**						
Any route (n=90)	1 (ref)	1·33 (0·83–2·13)	1·57 (1·01–2·43)	1·19 (0·73–1·94)	1·37 (0·88–2·11)	0·067
Smoking (n=62)	1 (ref)	1·53 (0·93–2·53)	1·63 (1·00–2·66)	0·93 (0·50–1·72)	1·27 (0·75–2·16)	0·215
Ingestion (n=34)	1 (ref)	0·75 (0·30–1·83)	1·42 (0·74–2·70)	1·33 (0·67–2·62)	1·16 (0·60–2·23)	0·320
**Lung cancer (n=116)**						
Any route (n=57)	1 (ref)	1·15 (0·49–2·73)	2·34 (1·23–4·43)	2·04 (1·05–3·95)	3·19 (1·85–5·50)	<0·0001
Smoking (n=37)	1 (ref)	0·40 (0·09–1·64)	1·16 (0·50–2·72)	1·71 (0·86–3·38)	2·73 (1·57–4·75)	0·0006
Ingestion (n=27)	1 (ref)	2·70 (1·23–5·93)	2·74 (1·30–5·79)	1·35 (0·48–3·74)	1·85 (0·85–4·02)	0·024
**Colon cancer (n=95)**						
Any route (n=15)	1 (ref)	1·58 (0·71–3·51)	0·49 (0·11–2·06)	0·74 (0·22–2·44)	0·66 (0·19–2·25)	0·379
Smoking (n=10)	1 (ref)	1·75 (0·75–4·11)	..	0·92 (0·28–3·00)	0·33 (0·04–2·47)	0·226
Ingestion (n=5)	1 (ref)	0·73 (0·10–5·31)	1·49 (0·35–6·18)	..	1·47 (0·34–6·27)	0·994
**Brain cancer (n=80)**						
Any route (n=17)	1 (ref)	0·28 (0·03–2·05)	2·08 (0·95–4·58)	0·83 (0·25–2·76)	1·33 (0·48–3·63)	0·476
Smoking (n=8)	1 (ref)	0·32 (0·04–2·37)	0·95 (0·29–3·11)	0·59 (0·14–2·49)	0·68 (0·16–2·94)	0·383
Ingestion (n=10)	1 (ref)	..	3·88 (1·51–9·94)	1·89 (0·45–7·97)	2·70 (0·79–9·20)	0·017
**Pancreatic cancer (n=78)**						
Any route (n=22)	1 (ref)	0·91 (0·28–2·97)	1·50 (0·58–3·90)	1·19 (0·41–3·43)	2·66 (1·23–5·74)	0·028
Smoking (n=16)	1 (ref)	0·80 (0·19–3·31)	1·25 (0·38–4·10)	1·52 (0·53–4·31)	2·82 (1·21–6·60)	0·028
Ingestion (n=8)	1 (ref)	0·64 (0·08–4·69)	1·87 (0·57–6·13)	..	2·36 (0·81–6·87)	0·347
**Liver cancer (n=73)**						
Any route (n=20)	1 (ref)	1·08 (0·38–3·04)	0·76 (0·23–2·53)	1·25 (0·48–3·27)	1·76 (0·77–4·01)	0·254
Smoking (n=8)	1 (ref)	0·61 (0·14–2·53)	..	0·81 (0·25–2·65)	0·84 (0·25–2·79)	0·340
Ingestion (n=12)	1 (ref)	2·29 (0·70–7·44)	1·37 (0·32–5·75)	1·54 (0·36–6·50)	3·37 (1·27–8·92)	0·018
**Bladder cancer (n=47)**						
Any route (n=23)	1 (ref)	3·24 (1·28–8·20)	0·55 (0·07–4·21)	3·31 (1·27–8·59)	4·28 (1·81–10·15)	0·0009
Smoking (n=14)	1 (ref)	2·61 (0·99–6·87)	..	1·53 (0·45–5·16)	2·62 (1·01–6·80)	0·107
Ingestion (n=10)	1 (ref)	1·05 (0·14–7·84)	2·83 (0·84–9·52)	2·05 (0·47–8·84)	3·12 (1·01–9·60)	0·018
**Laryngeal cancer (n=38)**						
Any route (n=23)	1 (ref)	1·11 (0·24–5·01)	2·55 (0·87–7·42)	2·98 (1·08–8·22)	3·34 (1·33–8·34)	0·0004
Smoking (n=16)	1 (ref)	..	2·19 (0·73–6·55)	1·99 (0·66–6·01)	3·15 (1·30–7·58)	0·0006
Ingestion (n=9)	1 (ref)	0·87 (0·11–6·52)	2·31 (0·68–7·79)	0·79 (0·10–5·94)	2·08 (0·68–6·38)	0·206

Data are adjusted hazard ratio (95% CI), unless otherwise indicated. This model used age as the timescale and was adjusted for sex (male *vs* female), ethnicity (Turkman *vs* non-Turkman), residence (urban *vs* rural), wealth score quartiles, smoking cigarettes (ever *vs* never), cumulative pack-years of smoked cigarettes (continuous variable), and regular alcohol drinking (never *vs* ever). For participants who used opium via both routes, we calculated the amount of opium used through each route separately; therefore, the numbers in each category of smoking and ingestion routes do not sum to the number of any route because these categories also include the few dual-route opium users. The amount of opium was calculated as nokhod-years. In each quartile, the cumulative opium used through the presented routes was as follows: any route, first quartile (≤5), second quartile (5·1–21), third quartile (21·1–60), and fourth quartile (>60); opium smoking, first quartile (≤4), second quartile (4·1–18), third quartile (18·1–60), and fourth quartile (>60); and opium ingestion, first quartile (≤9), second quartile (9·1–30), third quartile (30·1–78), and fourth quartile (>78).

When we assessed site-specific cancers, use of opium was associated with an increased risk of developing oesophageal (HR 1·38, 95% CI 1·06–1·80), gastric (1·36, 1·03–1·79), lung (2·21, 1·44–3·39), bladder (2·86, 1·47–5·55), and laryngeal (2·53, 1·21–5·30) cancers (figure 1) in a dose–response manner (figure 1; table 3). High-dose opium use was further associated with pancreatic cancer (2·66, 1·23–5·74; table 3). Most of these associations were observed among current opium users, but not in former opium users, although the statistical power for these comparisons was modest because of the small numbers of former opium users in the cohort (appendix p 8).

When we compared the route of opium use, we observed similar associations between both ingesting and smoking opium with all cancers combined (ingesting HR 1·49, 1·25–1·78; smoking 1·32, 1·15–1·52), gastrointestinal cancers combined (ingesting 1·33, 1·04–1·70; smoking 1·28, 1·05–1·56), respiratory cancers combined (ingesting 2·61, 1·60–4·26; smoking 2·05, 1·35–3·10), lung cancer (ingesting 2·66, 1·51–4·68; smoking 1·90, 1·17–3·10), bladder cancer (ingesting 3·79, 1·61–8·88; smoking 2·56, 1·21–5·40), and laryngeal cancer (ingesting 2·54, 1·14–5·68; smoking 2·48, 0·93–6·62). By contrast, associations were stronger for opium smoking with oesophageal cancer (1·43, 1·04–1·95) and gastric cancer (1·41, 1·03–1·93), whereas associations for opium ingestion were stronger with brain cancer (2·15, 1·00–4·63) and liver cancer (2·46, 1·23–4·95; table 4; appendix p 9). Most of the associations were dose-dependent (table 3). Associations between opium use and other less common cancers are shown in the appendix (pp 10–11).

**Table 4:** Routes of using opium and risk of different cancer types

		**Never used opium (n=41 548)**	**Only smoked opium (n=5810)**	**Only ingested opium (n=2156)**	**Both routes (n=520)**
**All cancers combined**					
n (%)		1351 (73·7%)	281 (15·3%)	160 (8·7%)	41 (2·2%)
Entire cohort (n=1833)*		1 (ref)	1·32 (1·15–1·52)	1·49 (1·25–1·78)	1·71 (1·24–2·36)
	Never used tobacco (n=1361)†	1 (ref)	1·34 (1·11–1·61)	1·24 (0·94–1·63)	1·68 (0·95–2·98)
	Ever used tobacco (n=472)‡	1 (ref)	1·32 (1·06–1·64)	1·79 (1·39–2·30)	1·76 (1·18–2·64)
**Gastrointestinal cancers combined**					
n (%)		672 (73·5%)	143 (15·6%)	80 (8·8%)	19 (2·1%)
Entire cohort (n=914)*		1 (ref)	1·28 (1·05–1·56)	1·33 (1·04–1·70)	1·48 (0·93–2·37)
	Never used tobacco (n=685)†	1 (ref)	1·35 (1·05–1·74)	0·95 (0·63–1·42)	2·08 (1·07–4·03)
	Ever used tobacco (n=229)‡	1 (ref)	1·18 (0·86–1·62)	1·71 (1·21–2·43)	1·18 (0·60–2·29)
**Respiratory cancers combined**					
n (%)		74 (48·1%)	44 (28·6%)	27 (17·5%)	9 (5·8%)
Entire cohort (n=154)*		1 (ref)	2·05 (1·35–3·10)	2·61 (1·60–4·26)	3·08 (1·46–6·49)
	Never used tobacco (n=59)†	1 (ref)	2·16 (1·00–4·65)	1·69 (0·51–5·53)	3·36 (0·45–24·69)
	Ever used tobacco (n=95)‡	1 (ref)	2·06 (1·24–3·44)	2·84 (1·60–5·04)	3·02 (1·32–6·91)
**Oesophageal cancer**					
n (%)		249 (72·8%)	55 (16·1%)	29 (8·5%)	9 (2·6%)
Entire cohort (n=342)*		1 (ref)	1·43 (1·04–1·95)	1·20 (0·79–1·82)	1·95 (0·98–3·87)
	Never used tobacco (n=266)†	1 (ref)	1·58 (1·08–2·30)	0·90 (0·47–1·69)	2·34 (0·86–6·31)
	Ever used tobacco (n=76)‡	1 (ref)	1·19 (0·69–2·07)	1·57 (0·85–2·89)	1·69 (0·64–4·46)
**Gastric cancer**					
n (%)		218 (70·8%)	56 (18·2%)	28 (9·1%)	6 (1·9%)
Entire cohort (n=308)*		1 (ref)	1·41 (1·03–1·93)	1·30 (0·83–1·97)	1·28 (0·54–2·92)
	Never used tobacco (n=255)†	1 (ref)	1·39 (0·92–2·11)	0·96 (0·49–1·90)	1·21 (0·30–4·92)
	Ever used tobacco (n=83)‡	1 (ref)	1·53 (0·91–2·57)	1·65 (0·91–2·99)	1·36 (0·47–3·93)
**Lung cancer**					
n (%)		59 (50·9%)	30 (25·9%)	20 (17·2%)	7 (6·0%)
Entire cohort (n=116)*		1 (ref)	1·90 (1·17–3·10)	2·66 (1·51–4·68)	3·27 (1·40–4·64)
	Never used tobacco (n=49)†	1 (ref)	1·84 (0·77–4·41)	1·27 (0·30–5·36)	..
	Ever used tobacco (n=67)‡	1 (ref)	2·12 (1·13–3·96)	3·37 (1·70–6·69)	4·13 (1·65–10·37)
**Colon cancer**					
n (%)		80 (84·2%)	10 (10·5%)	5 (5·3%)	0
Entire cohort (n=95)*		1 (ref)	0·88 (0·43–1·79)	1·14 (0·44–2·96)	..
	Never used tobacco (n=75)†	1 (ref)	1·03 (0·40–2·61)	1·04 (0·26–4·33)	..
	Ever used tobacco (n=20)‡	1 (ref)	0·66 (0·22–1·93)	1·26 (0·33–4·77)	..
**Brain cancer**					
n (%)		64 (80·0%)	7 (8·8%)	9 (11·3%)	1 (1·3%)
Entire cohort (n=80)*		1 (ref)	0·71 (0·31–1·64)	2·15 (1·00–4·63)	1·05 (0·14–7·90)
	Never used tobacco (n=61)†	1 (ref)	0·97 (0·35–2·72)	1·76 (0·54–5·72)	..
	Ever used tobacco (n=19)‡	1 (ref)	0·49 (0·13–1·86)	2·45 (0·82–7·34)	1·40 (0·17–11·50)
**Pancreatic cancer**					
n (%)		47 (70·1%)	12 (17·9%)	6 (8·9%)	2 (2·9%)
Entire cohort (n=78)*		1 (ref)	1·60 (0·84–3·05)	1·34 (0·54–3·28)	1·99 (0·46–8·54)
	Never used tobacco (n=56)†	1 (ref)	1·63 (0·68–3·87)	0·54 (0·07–3·98)	3·34 (0·45–24·45)
	Ever used tobacco (n=22)‡	1 (ref)	1·67 (0·61–4·59)	2·09 (0·65–6·77)	1·57 (0·18–13·03)
**Liver cancer**					
n (%)		53 (72·6%)	8 (11·0%)	12 (16·4%)	0
Entire cohort (n=73)*		1 (ref)	0·78 (0·35–1·71)	2·46 (1·23–4·95)	..
	Never used tobacco (n=49)†	1 (ref)	1·09 (0·38–3·09)	1·80 (0·54–5·92)	..
	Ever used tobacco (n=24)‡	1 (ref)	0·48 (0·15–1·54)	2·50 (0·99–6·35)	..
**Bladder cancer**					
n (%)		24 (51·0%)	13 (27·7%)	9 (19·1%)	1 (2·1%)
Entire cohort (n=47)*		1 (ref)	2·56 (1·21–5·40)	3·79 (1·61–8·88)	1·66 (0·21–13·02)
	Never used tobacco (n=26)†	1 (ref)	3·22 (1·15–9·01)	6·27 (2·01–19·55)	..
	Ever used tobacco (n=21)‡	1 (ref)	1·78 (0·62–5·04)	2·13 (0·64–7·06)	1·48 (0·17–12·66)
**Laryngeal cancer**					
n (%)		15 (39·5%)	14 (36·8%)	7 (18·4%)	2 (5·3%)
Entire cohort (n=38)*		1 (ref)	2·54 (1·14–5·68)	2·48 (0·93–6·62)	2·61 (0·55–12·41)
	Never used tobacco (n=10)†	1 (ref)	4·52 (0·86–23·67)	4·92 (0·55–44·04)	31·03 (3·44–279·31)
	Ever used tobacco (n=28)‡	1 (ref)	1·94 (0·80–4·71)	1·84 (0·63–5·37)	1·00 (0·12–8·36)

Data are hazard ratio (95% CI), unless otherwise indicated.

* This model used age as the timescale and was adjusted for sex, ethnicity (Turkman *vs* non-Turkman), residence (urban *vs* rural), wealth score quartiles, smoking cigarettes (ever *vs* never), cumulative pack-years of smoked cigarettes (continuous variable), and regular alcohol drinking (never *vs* ever).

† This model used age as the timescale and was adjusted for sex, ethnicity (Turkman *vs* non-Turkman), residence (urban *vs* rural), wealth score quartiles, and regular alcohol drinking (never *vs* ever).

‡ This model used age as the timescale and was adjusted for sex, ethnicity (Turkman *vs* non-Turkman), residence (urban *vs* rural), wealth score quartiles, cumulative pack-years of smoked cigarettes (continuous variable), and regular alcohol drinking (never *vs* ever).

All types of opium derivatives used in the cohort appeared to be associated with cancer incidence, although 7306 (86·0%) of 8486 opium users in the cohort used raw opium and very few users used other types, such as heroin or opium dross (appendix pp 12–13).

Stratification of the analyses by tobacco use (figure 1; table 4), socioeconomic status (appendix pp 14–15), and sex (appendix pp 16–17) revealed similar results across strata. Similarly, the results remained consistent after exclusion of the first 2 years of follow-up (appendix pp 18–19) and after exclusion of cancer cases who did not have histological confirmation (appendix pp 20–21).

## Discussion

In the GCS, opium use was associated with a higher risk of multiple cancers occurring in the respiratory, digestive, and urinary tract and CNS. Both ingesting and smoking opium were associated with cancer. Consistent associations were observed among ever and never tobacco users, men and women, and individuals with lower and higher socioeconomic status.

Within the gastrointestinal system, opium was associated with a dose-dependent increase in the risk of developing oesophageal, gastric, pancreatic, and liver cancers. Our results are supported by previous, albeit scarce, studies. The relationship between opium use and oesophageal cancer was first suggested in the 1970s,^2^, ^13^ and since then several case-control studies in Iran have documented this association.^2^, ^3^ Previous analyses from the GCS also indicated a higher risk of oesophageal cancer among opium users.^20^, ^21^ Consistent with our findings, two case-control studies and another cohort study also showed a higher risk of gastric cancer among opium users.^2^, ^4^, ^5^ To our knowledge, only two studies evaluated the relationship between opium use and pancreatic cancer—a case-control study^6^ and a previous analysis from the GCS^22^ with fewer cases and shorter follow-up duration—and both studies showed an increased risk of pancreatic cancer among opium users. Despite evidence in animal studies that opiates might play a part in the initiation and progression of liver cancer,^14^, ^15^ to our knowledge only one human study has assessed this outcome and reported higher liver cancer mortality among opioid-dependent individuals.^23^ In contrast to some case-control studies,^24^ we did not find any association between opium use and colon cancer.

We also observed associations between opium use and non-gastrointestinal cancers, including lung, laryngeal, bladder, and brain cancers. The relationship between opium use and bladder cancer was suggested in the 1970s, and since then has been shown in several case-control studies.^2^, ^11^ The increased risk of respiratory malignancies in opium users was also suggested in the 1970s by two case-control studies in Singapore and Hong Kong that showed an increased risk of lung cancer^9^ and laryngeal cancer^8^ among opium users. Several other case-control studies have also shown increased risks of laryngeal^7^ and lung^10^ cancers among opium users. Additionally, previous analyses from the GCS with shorter follow-up^12^, ^25^ and an Australian cohort of opioid-dependent individuals^23^ showed an increased risk of death due to malignant respiratory diseases, among opium-dependent or opioid-dependent individuals. To our knowledge, our study is the first in humans that suggests a link between use of opium and brain cancer, which is consistent with evidence from in-vitro studies indicating the presence of opiates and their receptors in brain tumour cells, and suggesting a possible role of opiates in tumour proliferation.^16^, ^17^

Three mechanisms have been proposed for the causal association between opium use and cancer. The first potential mechanism is the genotoxic or mutagenic effect of opium smoke and pyrolysates, and some opium alkaloids.^13^, ^26^ During opium pyrolysis, multiple carcinogenic compounds are produced including heterocyclic and polycyclic aromatic hydrocarbons, primary aromatic amines, and N-nitrosamines, which can enter the body through the respiratory and digestive tracts and affect different organs.^13^ Additionally, experimental studies have shown several types of chromosomal damage after exposure to different opiates.^26^ Furthermore, exposure to opium pyrolysates and morphine can cause a dose–response increase in the mutation frequencies of bacteria and human lymphocytes.^13^, ^26^, ^27^ The second potential mechanism is through the tumour promoting effects of opiates.^28^ Opiates have been shown to activate angiogenesis and neovascularisation,^28^ facilitate cancer cell proliferation and migration,^28^ and impair immune functions.^29^ The third potential mechanism is through facilitation of the effects of other carcinogens on different tissues, either by modifying the pharmacokinetics of these carcinogens and increasing their bioavailability^30^ or by impairing the physiological function of some organs and thus prolonging their exposure to the potential carcinogens.^2^

The current study shows that both opium smoking and opium ingestion can increase cancer risk in different organs. Furthermore, we previously showed that both routes of opium use can increase the risk of deaths due to circulatory, respiratory, digestive, and infectious diseases.^12^, ^20^, ^25^ Whereas smoking opium exposes individuals to higher levels of heterocyclic and polycyclic aromatic hydrocarbons and aromatic amines, ingesting opium exposes individuals to higher levels of morphine and other alkaloids.^12^, ^13^ We analysed urinary biomarkers of polycyclic aromatic hydrocarbons and volatile organic compounds in a group of GCS participants and found significantly higher concentrations of these biomarkers among both opium smokers and those who ingested opium compared with non-opium users (unpublished).

The strengths of this study are the large sample size, long follow-up period with less than 1% loss to follow-up, little missing data, presence of a uniquely large group of regular opium users, validation of self-reported opium consumption using urinary biomarkers, stringent sensitivity analyses (including among never tobacco users), and relatively low prevalence of important confounders such as alcohol and tobacco (especially in women).

This study also has some limitations. Like any observational study, we cannot rule out potential errors in exposure and outcome measurements. However, because of the prospective design of this study, any errors in measuring the exposure are likely to be non-differential differences in potential exposure errors between those who developed the studied outcomes and those who did not develop any of the studied outcomes. Furthermore, to minimise the possibility of outcome measurement errors we followed a strict approach towards case verification using several independent sources and did a sensitivity analysis restricted to histologically confirmed cases. In this study, we did not test the contents of the opium used, and therefore might have missed the presence of contaminants (including lead), which could have contributed to carcinogenicity. However, observing the relationship between opium use and cancers in different populations, documentation of different carcinogenic mechanisms for opiates in experimental studies, and observing the increased cancer risk with all routes and types of used opium make it unlikely that the effects were due to contaminations. Although our study shows higher cancer risk among opium smokers and those who ingest opium compared with those who have never used opium, caution is required in interpreting these results, as low statistical power for some cancer types prevents discrimination between the risks of the two routes of administration. Finally, despite adjustments for potential confounders and different sensitivity analyses, we cannot exclude the possibility of residual confounding, particularly for some cancer types that showed a modest risk increase associated with opium use. Also, the small number of cases in some cancer types and analytic strata might have resulted in spurious associations or unstable results and therefore requires investigation in further studies.

In conclusion, regular use of opium might be associated with increased cancer risk in multiple sites of the respiratory, digestive, urinary, and central nervous systems, among ever and never tobacco users, men and women, and individuals with lower and higher socioeconomic status. Given the recent increase in using opium derivatives, further global initiatives to reduce the misuse and implement preventive strategies to mitigate hazardous long-term effects are needed.
