# Supplementary material for: Opium use and subsequent incidence of cancer: results from the Golestan Cohort Study
Source: Lancet Glob Health. 2020 Apr 27;8(5):e649–60. doi: 10.1016/S2214-109X(20)30059-0 (PMC7196888; doi:10.1016/S2214-109X(20)30059-0)
Supplement: Supplementary appendix [file mmc1.pdf]

THE LANCET
Global Health

Supplementary appendix

This appendix formed part of the original submission and has been peer reviewed. We post it as supplied by the authors.

Supplement to: Sheikh M, Shakeri R, Poustchi H, et al. Opium use and subsequent incidence of cancer: results from the Golestan Cohort Study. *Lancet Glob Health* 2020; **8:** e649–60.

Supplementary File 1

Details of the interviews [1]

Each subject was interviewed by a trained general physician and a trained nutritionist, either in the local language (Turkmen) or in the national formal language (Persian), depending on the participant's preference. Two structured questionnaires were administered: a life-style questionnaire and a food frequency questionnaire (FFQ). Following the questionnaires and a limited physical examination, samples of blood, urine, hair, and nails were collected by a trained technician.

Validity and reliability of the questionnaires [2,3]

In the pilot phase of the Golestan Cohort Study 1,057 were interviewed, and a repeat interview was performed on 131 subjects 2 months after the first interview. The kappa statistics for agreement were above 0.7 for most variables, including tobacco, nass, opium, and alcohol consumption. The validity of the questionnaire data about opium use was assessed in 150 subjects by comparing their questionnaire responses with the presence of codeine or morphine in their urine; the questionnaire responses had a sensitivity of 0.93 and a specificity of 0.89 for identifying subjects with these urinary opium metabolites. There was also a good agreement between self-reported current tobacco smoking or nass use and positive urinary cotinine. To validate the study FFQ, twelve 24-h recall questionnaires (one every month) and four FFQs (one in each season) were administered to 131 participants during 1 year. There was good correlation between FFQ and recall data on food group and nutrient intakes, and there was acceptable correlation between FFQ data and biomarker measurements.

To examine the repeatability of the data collected in the actual cohort, we repeated the entire enrollment process, including interviews and sample collections, in 698 cohort participants from rural areas. The mean interval between the first and second enrollments was 45 months. The results showed very good agreement between data collected at the two interviews.

Methods of creating the wealth score [4]

We have previously published the details of the methods we used for creating the wealth score for GCS participants [4]. Briefly, to build a composite score for wealth based on appliances and other variables, we utilized multiple correspondence analysis (MCA) on personal car, motorbike, B/W TV, colour TV, refrigerator, freezer, vacuum and washing machine ownership variables, as well as house ownership, house structure, house size (tertiles), having a bath in the residence and occupation. MCA may be used as an exploratory tool and is appropriate for qualitative variables.

Brief description of multiple correspondence analysis used for creating the wealth score [4]

MCA is a technique that may be used to gain insight into dependencies within data cross-classified by discrete factors. It has many similarities to principal components analysis (PCA), though the latter is designed for continuous variables. Since PCA is easier to understand, we briefly describe its use first. Then, we discuss correspondence analysis (CA), which is appropriate for data cross classified by two factors, and MCA. PCA constructs a set of linear combinations of variables with the following properties. The first principal component is the linear combination of the variables that has the greatest variance amongst all the linear combinations in that model. The second principal component is the combination that has the second greatest proportion, and is orthogonal to the first, and so on for the third, fourth, and more principal components. The output is a set of weights which define the set of linear combinations. The aim is often to find a small number of linear combinations that explain a large proportion of the variability in the data. Technically, the weights and the proportion of variation accounted for are obtained by carrying out a singular value decomposition of the variance-covariance matrix of the data, to produce the eigenvalues and eigenvectors.

The basic approach in CA is similar, though the discreteness of the variables suggests that the variance-covariance matrix is not an appropriate quantity to decompose. Instead, the inertia, which is a scaling of the Pearson's chi-squared statistic, is examined. The inertia is a linear combination of elements that measure the distance of each contingency cell entry from independence, and the matrix of such entries are decomposed. In

MCA, a two-way table may be constructed by cross classifying the data by the levels of each of the variables. Again, in a similar fashion to PCA, one may plot the weights of the first combinations in order to see the contributions of each variable to the scores. In our study, we plot the first two combinations. The weights given to each level of each of the variables in each linear combination may be used to construct an index.. We used weights in the first linear combination (illustrated as the first dimension in Figure 1) to produce our wealth score, since it explained the majority of the chi-square variation of the wealth-related variables. For example, the weights for owning or not owning a refrigerator were 0.038 and – 0.733, and for owning or not owning a freezer were 0.542 and – 0.105, respectively. If a subject owned a refrigerator but did not have a freezer, the corresponding weights (0.038 and – 0.105) were summed up; this procedure was continued until the weights of all MCA variables were included in this calculation. More information about MCA is available in Encyclopedia of Statistical Sciences.

Methods for creating the Healthy Eating Index (HEI) dietary score [5]

We have previously published the details of the methods we used for creating the HEI dietary score for GCS participants [5]. To create components of the HEI, we converted the daily intakes from grams to cup and ounce equivalents using the Food Patterns Equivalents Database (FPED) 2013–2014 [6]. The FPED units are ounce and cup equivalents and can be converted to standard units as follows: 1 oz=28.35 g and 1 cup=225 ml. For fruits and vegetables, we used an extensive list of one cup equivalent weights for fruits and vegetables in the FPED [6]. For example, for canned fruit in light syrup, 65% fruit was assumed. For grain products such as bread, dough and cake, made with four, each 16 g of four present in a food was used as the basis for defning a 1-oz grain equivalent, the rationale being that one standard slice of bread has been defned as equal to 1-oz grain equivalent, which will contain 16 g of four [6]. For intact grains such as rice and pasta, cooked grains were converted to the uncooked forms with conversion factors 0.36 and 0.37, respectively [7], and 1-oz equivalent of grains was defned as 28.35 g [6]. In the FFQ, multi-ingredient foods such as pizza were not asked, so we did not have to disaggregate the foods. However, protein foods were further disaggregated to lean fraction and fat as follows: meat and poultry were disaggregated to lean meat and solid fat fractions; and seafood and nuts were disaggregated to lean protein and oil fractions. Similarly, dairy foods were further disaggregated to a low fat dairy fraction, similar to skim milk, and a solid fat fraction [6].

References

1 Pourshams A, Khademi H, Malekshah AF, *et al.* Cohort Profile: The Golestan Cohort Study--a prospective study of oesophageal cancer in northern Iran. *Int J Epidemiol* 2010; **39**: 52–9.

2 Pourshams A, Saadatian-Elahi M, Nouraie M, *et al.* Golestan cohort study of oesophageal cancer: feasibility and first results. *Br J Cancer* 2005; **92**: 176–81.

3 Malekshah AF, Kimiagar M, Saadatian-Elahi M, *et al.* Validity and reliability of a new food frequency questionnaire compared to 24 h recalls and biochemical measurements: pilot phase of Golestan cohort study of esophageal cancer. *Eur J Clin Nutr* 2006; **60**: 971–7.

4 Islami F, Kamangar F, Nasrollahzadeh D, *et al.* Socio-economic status and oesophageal cancer: results from a population-based case–control study in a high-risk area. *Int J Epidemiol* 2009; **38**: 978–88.

5 Hashemian M, Farvid MS, Poustchi H, *et al.* The application of six dietary scores to a Middle Eastern population: a comparative analysis of mortality in a prospective study. *Eur J Epidemiol* 2019; **34**: 371–82.

6 Bowman SA, C.J., Friday JE, Lynch KL, and Moshfegh AJ. Food patterns equivalents database 2013–14: Methodology and User Guide. Food Surveys Research Group, Beltsville Human Nutrition Research Center, Agricultural Research Service, U.S. Department of Agriculture, Beltsville, Maryland. http://www.ars.usda.gov/nea/bhnrc/fsrg (accessed Sept 20, 2019).

7 Bowman SA, M.C., Carlson JL, Clemens JC, Lin B-H, and Moshfegh AJ. Food intakes converted to retail commodities databases: 2003–08: methodology and user guide, A.R.S. U.S. Department of Agriculture, Beltsville, MD, and U.S. Department of Agriculture, Economic Research Service, Editor. Washington, D.C. p. 48., 2013 https://data.nal.usda.gov/dataset/food-intakes-converted-retail-commodities-databases-ficrcd (accessed Sept 20, 2019).

Supplementary File 2

Dose-response associations, stratified by tobacco use status, between duration of opium use and risk of all cancers combined (Figures A & D), gastrointestinal cancers combined (Figures B & E), and respiratory cancers combined (Figures C & F).

The vertical axis shows the hazard ratios in a model that used age as the time-scale and was adjusted for sex, ethnicity (Turkman/non-Turkman), residence (urban/rural), wealth score quartiles, smoking cigarettes (ever/never), cumulative pack-years of smoked cigarettes (continuous variable), and regular alcohol drinking (never/ever).

1. Never Tobacco Users

A. All cancers combined (n=1,361)

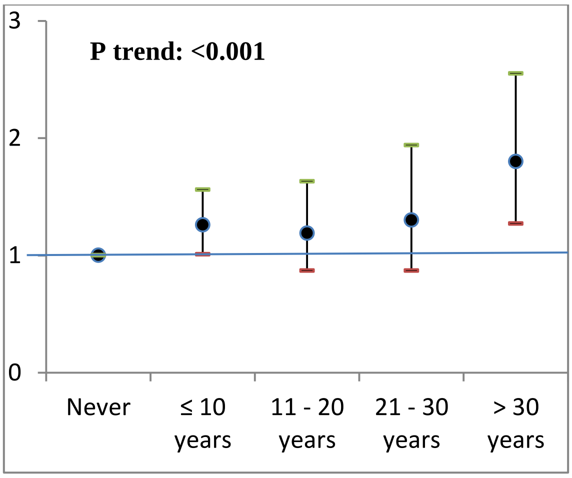

B. Gastrointestinal cancers combined (n=685)

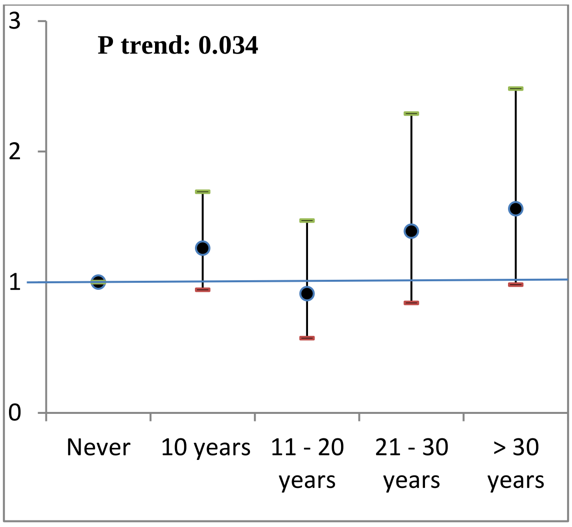

C. **Respiratory cancers combined (n=59)**

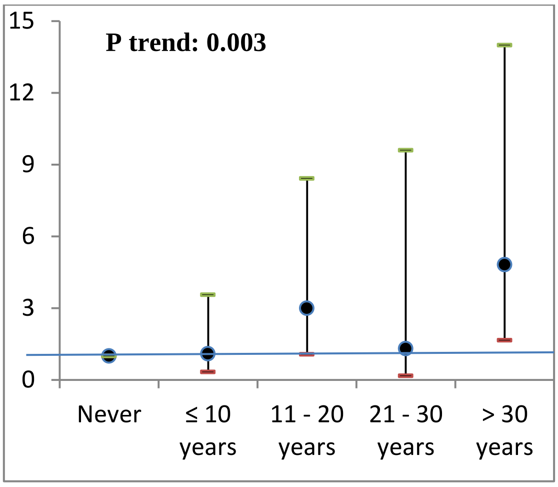

2. Ever Tobacco Users

D. **All cancers combined (n=472)**

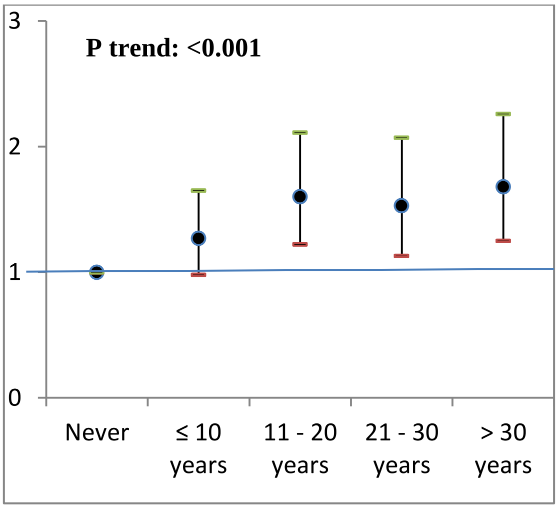

E. **Gastrointestinal cancers combined (n=229)**

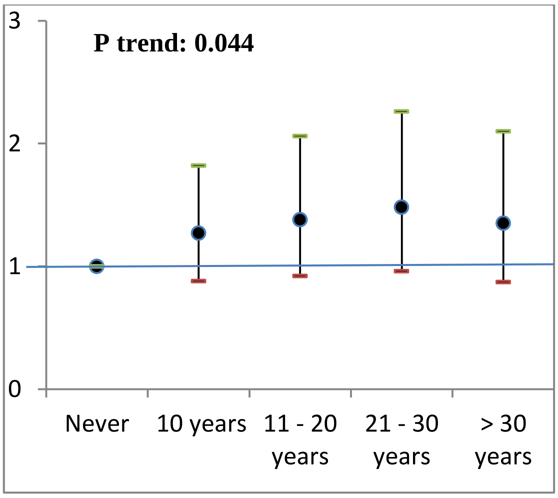

F. Respiratory cancers combined (n=95)

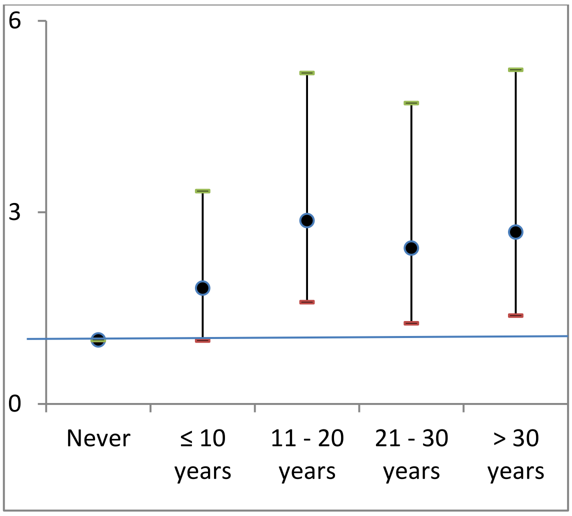

Supplementary Tables

Supplementary Table 1. Comparison of using two adjusted models for the evaluation of the effect of ever use of opium and risk of different cancer types in the Golestan Cohort Study.					
**Cancer Type**	**Case group n (%)**	**Non-case group n (%)**	**Unadjusted Model HR (95% CI)**	**Adjusted Model 1 ‡ HR (95% CI)**	**Adjusted Model 2 ¶ HR (95% CI)**
**All cancers combined (n=1,833)**					
• Never use of opium	1,351 (73.7)	40,197 (83.3)	1	1	1
• Ever use of opium	482 (26.3)	8,004 (16.6)	1.78 (1.60 – 1.97)	1.40 (1.24 – 1.58)	1.40 (1.23 – 1.59)
**Gastrointestinal cancers combined (n=914)**					
• Never use of opium	672 (73.5)	40,876 (83.2)	1	1	1
• Ever use of opium	242 (26.4)	8,244 (16.7)	1.77 (1.53 – 2.05)	1.31 (1.11 – 1.55)	1.32 (1.10 – 1.57)
**Respiratory cancers combined (n=154)**					
• Never use of opium	74 (48.0)	41,474 (83.1)	1	1	1
• Ever use of opium	80 (51.9)	8,406 (16.8)	5.40 (3.93 – 7.41)	2.28 (1.57 – 3.29)	2.25 (1.53 – 3.30)
**Esophageal cancer (n=342)**					
• Never use of opium	249 (72.8)	41,299 (83.1)	1	1	1
• Ever use of opium	93 (27.1)	8,393 (16.8)	1.83 (1.44 – 2.33)	1.38 (1.06 – 1.81)	1.36 (1.02 – 1.81)
**Gastric cancer (n=308)**					
• Never use of opium	218 (70.7)	41,330 (83.1)	1	1	1
• Ever use of opium	90 (29.2)	8,396 (16.8)	2.01 (1.57 – 2.57)	1.36 (1.04 – 1.80)	1.38 (1.02 – 1.85)
**Lung cancer (n=116)**					
• Never use of opium	59 (50.8)	41,489 (83.1)	1	1	1
• Ever use of opium	57 (49.1)	8,429 (16.8)	4.84 (3.36 – 6.96)	2.21 (1.44 – 3.38)	2.04 (1.31 – 3.19)
**Colon cancers (n=95)**					
• Never use of opium	80 (84.2)	41,468 (83.0)	1	1	1
• Ever use of opium	15 (15.7)	8,471 (16.9)	0.94 (0.54 – 1.63)	0.90 (0.48 – 1.66)	1.03 (0.55 – 1.94)
**Brain cancer (n=80)**					
• Never use of opium	63 (78.7)	41,485 (83.0)	1	1	1
• Ever use of opium	17 (20.9)	8,469 (16.9)	1.38 (0.80 – 2.35)	1.13 (0.61 – 2.09)	1.23 (0.66 – 2.29)
**Pancreatic cancer (n=78)**					
• Never use of opium	56 (71.7)	41,492 (83.0)	1	1	1
• Ever use of opium	22 (28.2)	8,464 (16.9)	1.95 (1.19 – 3.19)	1.54 (0.87 – 2.72)	1.49 (0.81 – 2.72)
**Liver cancer (n=73)**					
• Never use of opium	53 (72.6)	41,495 (83.0)	1	1	1
• Ever use of opium	20 (27.4)	8,466 (16.9)	1.88 (1.12 – 3.15)	1.22 (0.68 – 2.17)	1.21 (0.64 – 2.26)
**Bladder cancer (n=47)**					
• Never use of opium	24 (51.0)	41,524 (83.0)	1	1	1
• Ever use of opium	23 (48.9)	8,463 (16.9)	4.72 (2.66 – 8.37)	2.86 (1.47 – 5.53)	2.43 (1.20 – 4.93)
**Laryngeal cancer (n=38)**					
• Never use of opium	15 (39.4)	41,533 (83.0)	1	1	1
• Ever use of opium	23 (60.5)	8,463 (16.9)	7.61 (3.97 – 14.60)	2.53 (1.21 – 5.30)	2.99 (1.40 – 6.38)

Table footnotes:

HR: Hazards Ratio, CI: Confidence Interval, N: Number

‡: Adjusted Model 1 used age as the time-scale and was adjusted for sex, ethnicity (Turkman/non-Turkman), residence (urban/rural), wealth score quartiles, smoking cigarettes (ever/never), cumulative pack-years of smoked cigarettes (continuous variable), and regular alcohol drinking (never/ever)

¶: Adjusted Model 2 included all variables in model 1, and further included chewing nass (never/ two halves of nass-years), regular consumption of water pipe (never/ever), predominant household fuel (natural gas/kerosene/biomass/mixed), and tertiles of the healthy eating index

Supplementary Table2. Individual and combined effects of opium and tobacco use on the risk of developing different cancers				
**Cancer Type**	**Case group N (%)**	**Non-case group N (%)**	**Adjusted Model ‡ HR (95% CI)**	**P value for interaction**
**All cancers combined (n=1,833)**				
• Used neither opium nor tobacco	1,168	36,199	1	0.171
• Used opium but not tobacco	193	3,818	1.32 (1.13 – 1.54)	
• Used tobacco but not opium	183	3,998	1.17 (0.99 – 1.38)	
• Used both opium and tobacco	289	4,186	1.83 (1.58 – 2.12)	
**Gastrointestinal cancers combined (n=914)**				
• Used neither opium nor tobacco	580	3,906	1	0.505
• Used opium but not tobacco	105	3,818	1.27 (1.03 – 1.57)	
• Used tobacco but not opium	92	4,089	1.02 (0.80 – 1.29)	
• Used both opium and tobacco	137	4,338	1.46 (1.18 – 1.79)	
**Respiratory cancers combined (n=154)**				
• Used neither opium nor tobacco	47	37,320	1	0.471
• Used opium but not tobacco	12	3,999	1.94 (1.02 – 3.71)	
• Used tobacco but not opium	27	4,154	3.35 (1.96 – 5.72)	
• Used both opium and tobacco	68	4,407	8.71 (5.56 – 13.66)	
**Esophageal cancer (n=342)**				
• Used neither opium nor tobacco	220	37,147	1	0.979
• Used opium but not tobacco	46	3,965	1.41 (1.02 – 1.96)	
• Used tobacco but not opium	29	4,152	1.07 (0.71 – 1.62)	
• Used both opium and tobacco	47	4,428	1.51 (1.07 – 1.14)	
**Gastric cancer (n=308)**				
• Used neither opium nor tobacco	188	37,179	1	0.275
• Used opium but not tobacco	37	3,974	1.22 (0.85 – 1.75)	
• Used tobacco but not opium	30	4,151	0.79 (0.53 – 1.18)	
• Used both opium and tobacco	53	4,422	1.33 (0.96 – 1.86)	
**Lung cancer (n=116)**				
• Used neither opium nor tobacco	41	37,326	1	0.181
• Used opium but not tobacco	8	4,003	1.50 (0.69 – 3.25)	
• Used tobacco but not opium	18	4,163	2.56 (1.38 – 4.76)	
• Used both opium and tobacco	49	4,426	7.34 (4.43 – 12.13)	
**Colon cancer (n=95)**				
• Used neither opium nor tobacco	68	37,299	1	0.693
• Used opium but not tobacco	7	4,004	1.00 (0.45 – 2.22)	
• Used tobacco but not opium	12	4,169	1.13 (0.56 – 2.26)	
• Used both opium and tobacco	8	4,467	0.89 (0.40– 2.00)	
**Brain cancer (n=80)**				
• Used neither opium nor tobacco	54	37,313	1	0.811
• Used opium but not tobacco	7	4,004	1.21 (0.54 – 2.69)	
• Used tobacco but not opium	9	4,172	2.70 (1.17 – 6.19)	
• Used both opium and tobacco	10	4,465	2.83 (1.27 – 6.29)	
**Pancreatic cancer (n=78)**				
• Used neither opium nor tobacco	48	37,319	1	0.708
• Used opium but not tobacco	8	4,003	1.40 (0.65 – 3.00)	
• Used tobacco but not opium	8	4,173	1.44 (0.63 – 3.30)	
• Used both opium and tobacco	14	4,461	2.52 (1.25 – 5.07)	
**Liver cancer (n=73)**				
• Used neither opium nor tobacco	42	37,325	1	0.987
• Used opium but not tobacco	7	4,004	1.22 (0.54 – 2.75)	
• Used tobacco but not opium	11	4,170	1.66 (0.81 – 3.42)	
• Used both opium and tobacco	13	4,462	2.05 (1.03 – 4.07)	
**Bladder cancer (n=47)**				
• Used neither opium nor tobacco	17	37,350	1	0.341
• Used opium but not tobacco	9	4,002	3.74 (1.63 – 8.59)	
• Used tobacco but not opium	7	4,174	2.03 (0.78 – 5.27)	
• Used both opium and tobacco	14	4,461	4.21 (1.87 – 9.46)	
**Laryngeal cancer (n=38)**				
• Used neither opium nor tobacco	6	37,361	1	0.265
• Used opium but not tobacco	4	4,007	4.85 (1.33 – 17.62)	
• Used tobacco but not opium	9	4,172	8.65 (2.86 – 27.84)	
• Used both opium and tobacco	19	4,456	17.75 (6.06 – 51.94)	

Table footnotes:

HR: Hazards Ratio, CI: Confidence Interval, N: Number

‡: this model used age as the time-scale and was adjusted for sex, ethnicity (Turkman/non-Turkman), residence (urban/rural), wealth score quartiles, and regular alcohol drinking (never/ever)

Supplementary Table 3. Opium use status and risk of different cancer types in the Golestan Cohort Study.			
**Cancer Type**	**Cases group n (%)**	**Non-case group n (%)**	**Adjusted HR (95% CI) ‡**
**All cancers combined (n = 1,833)**			
• Never used opium	1,351 (73.7)	40,197 (83.3)	1
• Former opium user	44 (2.4)	824 (1.7)	1.04 (0.76 – 1.41)
• Current opium user	438 (23.9)	7,180 (14.9)	1.45 (1.28 – 1.64)
**Gastrointestinal cancers combined (n = 914)**			
• Never used opium	672 (73.5)	40,876 (83.2)	1
• Former opium user	26 (2.8)	842 (1.7)	1.17 (0.78 – 1.76)
• Current opium user	216 (23.6)	7,402 (15.0)	1.34 (1.13 – 1.59)
**Respiratory cancers combined (n=154)**			
• Never used opium	74 (48.0)	41,474 (83.1)	1
• Former opium user	7 (4.5)	861 (1.7)	1.53 (0.69 – 3.42)
• Current opium user	73 (47.4)	7,545 (15.1)	2.39 (1.64 – 3.47)
**Esophageal cancer (n = 342)**			
• Never used opium	249 (72.8)	41,299 (83.1)	1
• Former opium user	8 (2.3)	860 (1.7)	1.05 (0.51 – 2.16)
• Current opium user	85 (24.8)	7,533 (15.1)	1.44 (1.09 – 1.90)
**Gastric cancer (n = 308)**			
• Never used opium	218 (70.7)	41,330 (83.1)	1
• Former opium user	13 (4.2)	855 (1.7)	1.62 (0.91 – 2.89)
• Current opium user	77 (25.0)	7,541 (15.1)	1.34 (1.00 – 1.78)
**Lung cancer (n = 116)**			
• Never used opium	59 (50.8)	41,489 (83.1)	1
• Former opium user	4 (3.4)	864 (1.7)	1.21 (0.42 – 3.44)
• Current opium user	53 (45.6)	7,565 (15.1)	2.34 (1.52 – 3.60)
**Colon cancer (n = 95)**			
• Never used opium	80 (84.2)	41,468 (83.0)	1
• Former opium user	2 (2.1)	866 (1.7)	0.99 (0.23 – 4.17)
• Current opium user	13 (13.6)	7,605 (15.2)	0.89 (0.46 – 1.69)
**Brain cancer (n = 80)**			
• Never used opium	63 (78.7)	41,485 (83.0)	1
• Former opium user	2 (2.5)	866 (1.7)	1.02 (0.24 – 4.35)
• Current opium user	15 (18.7)	7,603 (15.2)	1.15 (0.60 – 2.17)
**Pancreatic cancer (n = 78)**			
• Never used opium	56 (71.7)	41,492 (83.0)	1
• Former opium user	3 (3.8)	865 (1.7)	1.76 (0.52 – 5.86)
• Current opium user	19 (24.3)	7,599 (15.2)	1.51 (0.84 – 2.73)
**Liver cancer (n = 73)**			
• Never used opium	53 (72.6)	41,495 (83.0)	1
• Former opium user	0	868 (1.7)	-
• Current opium user	20 (27.4)	7,598 (15.2)	1.39 (0.78 – 2.49)
**Bladder cancer (n = 47)**			
• Never used opium	24 (51.0)	41,524 (83.0)	1
• Former opium user	0	868 (1.7)	-
• Current opium user	23 (48.9)	7,595 (15.1)	3.36 (1.74 – 6.50)
**Laryngeal cancer (n = 38)**			
• Never used opium	15 (39.4)	41,533 (83.0)	1
• Former opium user	3 (7.8)	865 (1.7)	2.40 (0.65 – 8.80)
• Current opium user	20 (52.6)	7,598 (15.2)	2.55 (1.19 – 5.45)

Table footnotes:

HR: Hazards Ratio, CI: Confidence Interval, N: Number

‡: this model used age as the time-scale and was adjusted for sex, ethnicity (Turkman/non-Turkman), residence (urban/rural), wealth score quartiles, smoking cigarettes (ever/never), cumulative pack-years of smoked cigarettes (continuous variable), and regular alcohol drinking (never/ever)

**Supplementary Table 4. Subgroup analysis of gastric cancer, stratified by different subtypes of gastric cancers, on the route of opium use and risk of cancers in the Golestan Cohort Study.**				
**Cancer Type**	**Case group N (%)**	**Non-case group N (%)**	**Unadjusted Model HR (95% CI)**	**Adjusted Model ‡ HR (95% CI)**
**Cardia (n = 181)**				
**Ever use of opium**				
• Never used opium	133 (73.4)	41,415 (83.0)	1	1
• Ever use of opium	48 (26.5)	8,438 (16.9)	1.74 (1.25 – 2.42)	1.18 (0.81 – 1.70)
**Route of opium use**				
• Never used opium	133 (73.4)	41,415 (83.0)	1	1
• Only smoking	25 (13.8)	5,785 (11.6)	1.50 (0.98 – 2.31)	1.05 (0.67 – 1.66)
• Only ingestion	19 (10.5)	2,137 (4.2)	2.09 (1.29 – 3.39)	1.37 (0.82 – 2.29)
• Both smoking and ingestion	4 (2.2)	516 (1.0)	2.14 (0.79 – 5.80)	1.37 (0.49 – 3.81)
**Dose-response analysis (for the quartiles of the cumulative used opium)**				
• Any route	48	8,438	P for trend = <0.001	P for trend = 0.193
• Smoking	23	2,653	P for trend = 0.054	P for trend = 0.933
• Ingestion	29	6,294	P for trend = <0.001	P for trend = 0.082
**Non-cardia (n = 127)**				
**Ever use of opium**				
• Never used opium	85 (66.9)	41,463 (83.0)	1	1
• Ever use of opium	42 (33.0)	8,444 (16.9)	2.43 (1.68 – 3.52)	1.69 (1.11 – 2.56)
**Route of opium use**				
• Never used opium	85 (66.9)	41,463 (83.0)	1	1
• Only smoking	31 (24.4)	5,779 (11.5)	2.88 (1.91 – 4.35)	1.98 (1.26 – 3.11)
• Only ingestion	9 (7.0)	2,147 (4.3)	1.66 (0.83 – 3.31)	1.16 (0.56 – 2.38)
• Both smoking and ingestion	2 (1.5)	518 (1.0)	1.76 (0.43 – 7.16)	1.11 (0.26 – 4.66)
**Dose-response analysis (for the quartiles of the cumulative used opium)**				
• Any route	42	8,444	P for trend = <0.001	P for trend = 0.185
• Smoking	33	2,643	P for trend = <0.001	P for trend = 0.068
• Ingestion	11	6,312	P for trend = 0.420	P for trend = 0.569

Table footnotes:

HR: Hazards Ratio, CI: Confidence Interval, N: Number

‡: this model used age as the time-scale and was adjusted for sex, ethnicity (Turkman/non-Turkman), residence (urban/rural), wealth score quartiles, smoking cigarettes (ever/never), cumulative pack-years of smoked cigarettes (continuous variable), and regular alcohol drinking (never/ever)

Supplementary Table 5. Routes of using opium and risk of some less common cancer types in the Golestan Cohort Study.

Cancer Type	Case group N (%)	Non-case group N (%)	Unadjusted Model HR (95% CI)	Adjusted Model ‡ HR (95% CI)
**Breast cancer (n=86)**				
**Ever use of opium**				
• Never used opium	79 (91.8)	41,469 (83.0)	1	1
• Ever use of opium	7 (8.1)	8,479 (16.9)	0.48 (0.22 – 1.04)	1.55 (0.70 – 3.43)
**Route of opium use**				
• Never used opium	79 (91.8)	41,469 (83.0)	1	1
• Only smoking	5 (5.8)	5,805 (11.6)	0.46 (0.19 – 1.16)	1.53 (0.61 – 3.85)
• Only ingestion	2 (2.3)	2,154 (4.3)	0.64 (0.15 – 2.64)	1.85 (0.44 – 7.70)
• Both smoking and ingestion	0	520 (1.0)	-	-
**Leukemia (n=44)**				
**Ever use of opium**				
• Never used opium	31 (70.4)	41,517 (83.0)	1	1
• Ever use of opium	13 (29.5)	8,473 (16.9)	2.07 (1.08 – 3.95)	1.61 (0.78 – 3.33)
**Route of opium use**				
• Never used opium	31 (70.4)	41,517 (83.0)	1	1
• Only smoking	8 (18.1)	5,802 (11.6)	2.02 (0.93 – 4.41)	1.56 (0.68 – 3.60)
• Only ingestion	4 (9.0)	2,152 (4.3)	2.07 (0.72 – 5.91)	1.73 (0.57 – 5.18)
• Both smoking and ingestion	1 (2.2)	519 (1.0)	2.46 (0.33 – 18.06)	1.59 (0.19 – 12.85)
**Lymphoma (n=42)**				
**Ever use of opium**				
• Never used opium	32 (76.1)	41,516 (83.0)	1	1
• Ever use of opium	10 (23.8)	8,476 (16.9)	1.60 (0.78 – 3.26)	1.47 (0.67 – 3.20)
**Route of opium use**				
• Never used opium	32 (76.1)	41,516 (83.0)	1	1
• Only smoking	7 (16.6)	5,803 (11.6)	1.67 (0.73 – 3.78)	1.48 (0.62 – 3.56)
• Only ingestion	3 (7.1)	2,153 (4.3)	1.80 (0.54 – 5.92)	1.73 (0.50 – 5.98)
• Both smoking and ingestion	0	520 (1.0)	-	-
**Ovarian cancer (n=42)**				
**Ever use of opium**				
• Never used opium	40 (95.2)	41,508 (83.0)	1	1
• Ever use of opium	2 (4.7)	8,484 (16.9)	0.26 (0.06 – 1.08)	0.77 (0.18 – 3.27)
**Route of opium use**				
• Never used opium	40 (95.2)	41,508 (83.0)	1	1
• Only smoking	1 (2.3)	5,809 (11.6)	0.18 (0.02 – 1.35)	0.59 (0.08 – 4.35)
• Only ingestion	0	2,156 (4.3)	-	-
• Both smoking and ingestion	1 (2.3)	519 (1.0)	2.33 (0.32 – 16.99)	10.69 (1.44 – 79.20)
**Prostate cancer (n=32)**				
**Ever use of opium**				
• Never used opium	26 (81.2)	41,522 (83.0)	1	1
• Ever use of opium	6 (18.7)	8,480 (16.9)	1.09 (0.44 – 2.65)	0.62 (0.24 – 1.57)
**Route of opium use**				
• Never used opium	26 (81.2)	41,522 (83.0)	1	1
• Only smoking	3 (9.3)	5,807 (11.6)	0.95 (0.28 – 3.16)	0.54 (0.16 – 1.85)
• Only ingestion	3 (9.3)	2,153 (4.3)	1.53 (0.46 – 5.08)	0.88 (0.25 – 3.04)
• Both smoking and ingestion	0	520 (1.0)	-	-
**Oropharyngeal cancers (n=31)**				
**Ever use of opium**				
• Never used opium	22 (70.9)	41,526 (83.0)	1	1
• Ever use of opium	9 (29.0)	8,477 (16.9)	2.08 (0.96 – 4.54)	1.58 (0.65 – 3.81)
**Route of opium use**				
• Never used opium	22 (70.9)	41,526 (83.0)	1	1
• Only smoking	6 (19.3)	5,804 (11.6)	2.11 (0.85 – 5.21)	1.69 (0.58 – 4.49)
• Only ingestion	1 (3.2)	2,155 (4.3)	0.83 (0.11 – 6.21)	0.65 (0.08 – 5.05)
• Both smoking and ingestion	2 (6.4)	518 (1.0)	7.38 (1.73 – 31.53)	4.91 (1.00 – 24.03)
**Cervical cancer (n=25)**				
**Ever use of opium**				
• Never used opium	22 (88.0)	41,526 (83.0)	1	1
• Ever use of opium	3 (12.0)	8,483 (16.9)	0.72 (0.21 – 2.41)	1.46 (0.42 – 5.00)
**Route of opium use**				
• Never used opium	22 (88.0)	41,526 (83.0)	1	1
• Only smoking	0	5,810 (11.6)	-	-
• Only ingestion	3 (12.0)	2,153 (4.3)	3.22 (0.95 – 10.86)	5.11 (1.47 – 17.72)
	0	520 (1.0)	-	-

• Both smoking and ingestion				
**Rectal cancer (n=23)**				
**Ever use of opium**				
• Never used opium	18 (78.2)	41,530 (83.0)	1	1
• Ever use of opium	5 (21.7)	8,481 (16.9)	1.39 (0.51 – 3.77)	0.94 (0.32 – 2.74)
**Route of opium use**				
• Never used opium	18 (78.2)	41,530 (83.0)	1	1
• Only smoking	1 (4.3)	5,809 (11.6)	0.44 (0.04 – 2.30)	0.31 (0.04 – 2.39)
• Only ingestion	2 (8.7)	2,154 (4.3)	1.85 (0.30 – 5.89)	1.46 (0.31 – 6.76)
• Both smoking and ingestion	2 (8.7)	518 (1.0)	8.64 (1.29 – 25.05)	5.46 (1.08 – 27.43)

Table footnotes:

HR: Hazards Ratio, CI: Confidence Interval, N: Number

‡: this model used age as the time-scale and was adjusted for sex, ethnicity (Turkman/non-Turkman), residence (urban/rural), wealth score quartiles, smoking cigarettes (cumulative pack-years, continuous variable), and regular alcohol drinking (never/ever)

**Supplementary Table 6. The association between different types of opium and risk of cancers in the Golestan Cohort Study.**			
**Cancer Type**	**Case group n (%)**	**Non-case group n (%)**	**Adjusted Model ‡ HR (95% CI)**
**All cancers combined (n=1,833)**			
• Never used opium	1,351 (73.7)	40,197 (83.3)	1
• Raw opium (teriak)	415 (22.6)	6,891 (14.3)	1.40 (1.23 – 1.58)
• Refined opium (shireh)	36 (1.9)	746 (1.5)	1.18 (0.84 – 1.66)
• Burned opium (sukhteh)	1 (0.0)	5 (0.0)	7.46 (1.04 – 53.12)
• Heroin	1 (0.0)	3 (0.0)	14.64 (2.05 – 104.64)
• Combination of the above	29 (1.5)	359 (0.7)	1.67 (1.14 – 2.44)
**Gastrointestinal cancers combined (n=914)**			
• Never used opium	672 (73.5)	40,876 (83.2)	1
• Raw opium (teriak)	218 (23.8)	7,088 (14.4)	1.37 (1.15 – 1.62)
• Refined opium (shireh)	15 (1.6)	767 (1.5)	0.96 (0.57 – 1.62)
• Burned opium (sukhteh)	0	6 (0.0)	-
• Heroin	0	4 (0.0)	-
• Combination of the above	9 (0.9)	379 (0.7)	1.00 (0.51 – 1.95)
**Respiratory cancers combined (n=154)**			
• Never used opium	74 (48.0)	41,474 (83.1)	1
• Raw opium (teriak)	66 (42.8)	7,240 (14.5)	2.23 (1.53 – 3.27)
• Refined opium (shireh)	6 (3.9)	776 (1.5)	1.83 (0.77 – 4.33)
• Burned opium (sukhteh)	0	6 (0.0)	-
• Heroin	1 (0.6)	3 (0.0)	91.95 (12.03 – 702.70)
• Combination of the above	7 (4.5)	381 (0.7)	3.18 (1.40 – 7.21)
**Esophageal cancer (n=342)**			
• Never used opium	249 (72.8)	41,299 (83.1)	1
• Raw opium (teriak)	83 (24.2)	7,223 (14.5)	1.43 (1.09 – 1.89)
• Refined opium (shireh)	5 (1.4)	777 (1.5)	0.92 (0.37 – 2.26)
• Burned opium (sukhteh)	0	6 (0.0)	-
• Heroin	0	4 (0.0)	-
• Combination of the above	5 (1.4)	383 (0.7)	1.58 (0.64 – 3.93)
**Gastric cancer (n=308)**			
• Never used opium	218 (70.7)	41,330 (83.1)	1
• Raw opium (teriak)	81 (26.3)	7,225 (14.5)	1.42 (1.07 – 1.88)
• Refined opium (shireh)	7 (2.2)	775 (1.5)	1.26 (0.58 – 2.72)
• Burned opium (sukhteh)	0	6 (0.0)	-
• Heroin	0	4 (0.0)	-
• Combination of the above	2 (0.6)	386 (0.7)	0.61 (0.15 – 2.51)
**Lung cancer (n=116)**			
• Never used opium	59 (50.8)	41,489 (83.1)	1
• Raw opium (teriak)	48 (41.3)	7,258 (14.5)	2.19 (1.41 – 3.40)
• Refined opium (shireh)	3 (2.5)	779 (1.5)	1.25 (0.38 – 4.12)
• Burned opium (sukhteh)	0	6 (0.0)	-
• Heroin	1 (0.8)	3 (0.0)	109.28 (13.98 – 853.93)
• Combination of the above	5 (4.3)	383 (0.7)	3.05 (1.16 – 7.99)
**Colon cancer (n=95)**			
• Never used opium	80 (84.2)	41,468 (83.0)	1
• Raw opium (teriak)	14 (14.7)	7,292 (14.6)	0.97 (0.52 – 1.82)
• Refined opium (shireh)	1 (1.0)	781 (1.5)	0.64 (0.08 – 4.71)
• Burned opium (sukhteh)	0	6 (0.0)	-
• Heroin	0	4 (0.0)	-
• Combination of the above	0	388 (0.7)	-
**Brain cancer (n=80)**			
• Never used opium	63 (78.7)	41,485 (83.0)	1
• Raw opium (teriak)	15 (18.7)	7,291 (14.6)	1.17 (0.62 – 2.20)
• Refined opium (shireh)	0	782 (1.5)	-
• Burned opium (sukhteh)	0	6 (0.0)	-
• Heroin	0	4 (0.0)	-
• Combination of the above	2 (2.5)	386 (0.7)	2.80 (0.63 – 12.34)
**Pancreatic cancer (n=78)**			
• Never used opium	56 (71.7)	41,492 (83.0)	1
• Raw opium (teriak)	18 (23.0)	7,288 (14.5)	1.48 (0.81 – 2.68)
• Refined opium (shireh)	2 (2.5)	780 (1.5)	1.58 (0.37 – 6.73)
• Burned opium (sukhteh)	0	6 (0.0)	-
• Heroin	0	4 (0.0)	-
• Combination of the above	2 (2.5)	386 (0.7)	2.92 (0.67 – 12.73)
**Liver cancer (n=73)**			

• Never used opium	53 (72.6)	41,495 (83.0)	1
• Raw opium (teriak)	20 (27.4)	7,286 (14.5)	1.38 (0.77 – 2.46)
• Refined opium (shireh)	0	782 (1.5)	-
• Burned opium (sukhteh)	0	6 (0.0)	-
• Heroin	0	4 (0.0)	-
• Combination of the above	0	388 (0.7)	-
**Bladder cancer (n=47)**			
• Never used opium	24 (51.0)	41,524 (83.0)	1
• Raw opium (teriak)	17 (36.1)	7,289 (14.5)	2.51 (1.25 – 5.04)
• Refined opium (shireh)	4 (8.5)	778 (1.5)	5.80 (1.86 – 18.03)
• Burned opium (sukhteh)	0	6 (0.0)	-
• Heroin	0	4 (0.0)	-
• Combination of the above	2 (4.2)	386 (0.7)	5.00 (1.08 – 23.05)
**Laryngeal cancer (n=38)**			
• Never used opium	15 (39.4)	41,533 (83.0)	1
• Raw opium (teriak)	18 (47.3)	7,288 (14.5)	2.38 (1.10 – 5.12)
• Refined opium (shireh)	3 (7.8)	779 (1.5)	3.40 (0.92 – 12.55)
• Burned opium (sukhteh)	0	6 (0.0)	-
• Heroin	0	4 (0.0)	-
• Combination of the above	2 (5.2)	386 (0.7)	3.63 (0.77 – 17.15)

Table footnotes:

HR: Hazards Ratio, CI: Confidence Interval, N: Number

‡: this model used age as the time-scale and was adjusted for sex, ethnicity (Turkman/non-Turkman), residence (urban/rural), wealth score quartiles, smoking cigarettes (ever/never), cumulative pack-years of smoked cigarettes (continuous variable), and regular alcohol drinking (never/ever)

**Supplementary Table 7. Using opium and cancer risk analysis, stratified by socioeconomic status, in the Golestan Cohort Study.**			
**Cancer Type**	**Lower SES**	**Higher SES**	**P value for interaction**
	**Adjusted ‡ HR (95% CI)**	**Adjusted ‡ HR (95% CI)**	
**All cancers combined**	**n = 1,026**	**n = 807**	
**Ever use of opium**			0.693
• Never used opium<br>• Ever use of opium	1<br>1.41 (1.22 – 1.64)	1<br>1.37 (1.13 – 1.67)	
**Route of opium use**			
• Never used opium<br>• Only smoking<br>• Only ingestion<br>• Both smoking and ingestion	1<br>1.32 (1.10 – 1.58)<br>1.56 (1.26 – 1.92)<br>1.63 (1.07 – 2.46)	1<br>1.29 (1.03 – 1.61)<br>1.47 (1.06 – 2.04)<br>1.91 (1.14 – 3.18)	
**Gastrointestinal cancers combined**	**n = 552**	**n = 362**	
**Ever use of opium**			0.671
• Never used opium<br>• Ever use of opium	1<br>1.24 (1.01 – 1.53)	1<br>1.46 (1.10 – 1.94)	
**Route of opium use**			
• Never used opium<br>• Only smoking<br>• Only ingestion<br>• Both smoking and ingestion	1<br>1.15 (0.90 – 1.48)<br>1.40 (1.05 – 1.86)<br>1.29 (0.71 – 2.33)	1<br>1.54 (1.13 – 2.12)<br>1.13 (0.65 – 1.93)<br>1.92 (0.89 – 4.15)	
**Respiratory cancers combined**	**n = 81**	**n = 73**	
**Ever use of opium**			0.902
• Never used opium<br>• Ever use of opium	1<br>2.30 (1.39 – 3.80)	1<br>2.35 (1.36 – 4.03)	
**Route of opium use**			
• Never used opium<br>• Only smoking<br>• Only ingestion<br>• Both smoking and ingestion	1<br>2.09 (1.18 – 3.70)<br>2.41 (1.27 – 4.55)<br>3.63 (1.42 – 9.29)	1<br>2.07 (1.13 – 3.80)<br>3.04 (1.42 – 6.48)<br>2.78 (0.81 – 9.47)	
**Esophageal cancer**	**n = 242**	**n = 100**	
**Ever use of opium**			0.236
• Never used opium<br>• Ever use of opium	1<br>1.28 (0.93 – 1.76)	1<br>1.80 (1.07 – 3.01)	
**Route of opium use**			
• Never used opium<br>• Only smoking<br>• Only ingestion<br>• Both smoking and ingestion	1<br>1.22 (0.83 – 1.78)<br>1.38 (0.89 – 2.15)<br>1.36 (0.54 – 3.42)	1<br>2.15 (1.23 – 3.77)<br>0.50 (0.12 – 2.10)<br>4.13 (1.44 – 11.83)	
**Gastric cancer**	**n = 184**	**n = 124**	
**Ever use of opium**			0.798
• Never used opium<br>• Ever use of opium	1<br>1.24 (0.88 – 1.75)	1<br>1.61 (1.02 – 2.55)	
**Route of opium use**			
• Never used opium<br>• Only smoking<br>• Only ingestion<br>• Both smoking and ingestion	1<br>1.27 (0.85 – 1.90)<br>1.15 (0.69 – 1.91)<br>1.45 (0.58 – 3.65)	1<br>1.69 (1.01 – 2.82)<br>1.66 (0.77 – 3.54)<br>0.73 (0.10 – 5.37)	
**Lung cancer**	**n = 60**	**n = 56**	
**Ever use of opium**			0.691
• Never used opium<br>• Ever use of opium	1<br>2.25 (1.25 – 4.03)	1<br>2.21 (1.18 – 4.15)	
**Route of opium use**			
• Never used opium<br>• Only smoking<br>• Only ingestion<br>• Both smoking and ingestion	1<br>2.02 (1.04 – 3.93)<br>2.33 (1.10 – 4.92)<br>4.05 (1.41 – 11.56)	1<br>1.79 (0.87 – 3.69)<br>3.42 (1.46 – 8.03)<br>2.58 (0.58 – 11.43)	
**Colon cancer**	**n = 37**	**n = 58**	
**Ever use of opium**			0.384
• Never used opium<br>• Ever use of opium	1<br>1.28 (0.55 – 2.94)	1<br>0.60 (0.24 – 1.52)	
**Route of opium use**			
• Never used opium<br>• Only smoking<br>• Only ingestion<br>• Both smoking and ingestion	1<br>1.15 (0.42 – 3.16)<br>1.74 (0.56 – 5.35)<br>-	1<br>0.69 (0.25 – 1.84)<br>0.50 (0.06 – 3.77)<br>-	

**Brain cancer**	**n = 50**	**n = 30**	
**Ever use of opium**			0.877
• Never used opium<br>• Ever use of opium	1<br>1.27 (0.61 – 2.66)	1<br>0.95 (0.32 – 2.81)	
**Route of opium use**			
• Never used opium<br>• Only smoking<br>• Only ingestion<br>• Both smoking and ingestion	1<br>1.00 (0.40 – 2.53)<br>2.07 (0.81 – 5.30)<br>-	1<br>0.26 (0.03 – 2.10)<br>2.68 (0.72 – 9.92)<br>2.76 (0.34 – 22.05)	
**Pancreatic cancer**	**n = 42**	**n = 36**	
**Ever use of opium**			0.349
• Never used opium<br>• Ever use of opium	1<br>1.27 (0.58 – 2.75)	1<br>2.14 (0.91 – 4.99)	
**Route of opium use**			
• Never used opium<br>• Only smoking<br>• Only ingestion<br>• Both smoking and ingestion	1<br>1.36 (0.55 – 3.35)<br>1.11 (0.36 – 3.40)<br>1.42 (0.18 – 11.19)	1<br>2.02 (0.79 – 5.17)<br>2.17 (0.47 – 9.91)<br>3.53 (0.44 – 28.05)	
**Liver cancer**	**n = 43**	**n = 30**	
**Ever use of opium**			0.071
• Never used opium<br>• Ever use of opium	1<br>1.47 (0.73 – 1.96)	1<br>0.69 (0.19 – 2.47)	
**Route of opium use**			
• Never used opium<br>• Only smoking<br>• Only ingestion<br>• Both smoking and ingestion	1<br>0.72 (0.26 – 1.99)<br>3.28 (1.50 – 7.16)<br>-	1<br>0.98 (0.27 – 3.51)<br>-<br>-	
**Bladder cancer**	**n = 28**	**n = 19**	
**Ever use of opium**			0.553
• Never used opium<br>• Ever use of opium	1<br>3.42 (1.47 – 7.94)	1<br>2.20 (0.76 – 6.37)	
**Route of opium use**			
• Never used opium<br>• Only smoking<br>• Only ingestion<br>• Both smoking and ingestion	1<br>2.91 (1.11 – 7.61)<br>4.54 (1.61 – 12.74)<br>2.99 (0.36 – 24.84)	1<br>2.29 (0.72 – 7.33)<br>2.45 (0.50 – 12.08)<br>-	
**Laryngeal cancer**	**n = 21**	**n = 17**	
**Ever use of opium**			0.605
• Never used opium<br>• Ever use of opium	1<br>2.44 (0.91 – 6.56)	1<br>2.80 (0.93 – 8.40)	
**Route of opium use**			
• Never used opium<br>• Only smoking<br>• Only ingestion<br>• Both smoking and ingestion	1<br>2.32 (0.77 – 6.98)<br>2.66 (0.78 – 8.98)<br>2.42 (0.27 – 21.09)	1<br>3.02 (0.93 – 9.79)<br>2.14 (0.40 – 11.32)<br>3.31 (0.36 – 29.81)	

Table footnotes:

SES: Socioeconomic status

HR: Hazards Ratio, **CI:** Confidence Interval, **N:** Number

‡: this model used age as the time-scale and was adjusted for sex, ethnicity (Turkman/non-Turkman), residence (urban/rural), smoking cigarettes (ever/never), cumulative pack-years of smoked cigarettes (continuous variable), and regular alcohol drinking (never/ever)

Supplementary Table 8. Using opium and cancer risk analysis, stratified by gender, in the Golestan Cohort Study.			
Cancer Type	Male	Female	P value for interaction
	Adjusted ‡ HR (95% CI)	Adjusted ‡ HR (95% CI)	
**All cancers combined**	**n = 1,002**	**n = 831**	
**Ever use of opium**			0.437
• Never used opium<br>• Ever use of opium	1<br>1.43 (1.24 – 1.65)	1<br>1.26 (1.00 – 1.59)	
**Route of opium use**			
• Never used opium<br>• Only smoking<br>• Only ingestion<br>• Both smoking and ingestion	1<br>1.36 (1.16 – 1.60)<br>1.48 (1.20 – 1.82)<br>1.85 (1.31 – 2.60)	1<br>1.16 (0.87 – 1.55)<br>1.53 (1.09 – 2.14)<br>0.74 (0.23 – 2.33)	
**Gastrointestinal cancers combined**	**n = 560**	**n = 354**	
**Ever use of opium**			0.969
• Never used opium<br>• Ever use of opium	1<br>1.34 (1.10 – 1.62)	1<br>1.18 (0.83 – 1.66)	
**Route of opium use**			
• Never used opium<br>• Only smoking<br>• Only ingestion<br>• Both smoking and ingestion	1<br>1.30 (1.04 – 1.62)<br>1.35 (1.02 – 1.80)<br>1.66 (1.02 – 2.70)	1<br>1.17 (0.76 – 1.79)<br>1.29 (0.78 – 2.14)<br>0.47 (0.06 – 3.41)	
**Respiratory cancers combined**	**n = 122**	**n = 32**	
**Ever use of opium**			0.717
• Never used opium<br>• Ever use of opium	1<br>2.30 (1.54 – 3.44)	1<br>2.08 (0.74 – 5.83)	
**Route of opium use**			
• Never used opium<br>• Only smoking<br>• Only ingestion<br>• Both smoking and ingestion	1<br>2.18 (1.40 – 3.40)<br>2.37 (1.38 – 4.06)<br>3.20 (1.48 – 6.87)	1<br>1.03 (0.22 – 4.85)<br>4.57 (1.43 – 14.58)<br>-	
**Esophageal cancer**	**n = 185**	**n = 157**	
**Ever use of opium**			0.481
• Never used opium<br>• Ever use of opium	1<br>1.31 (0.94 – 1.82)	1<br>1.40 (0.87 – 2.23)	
**Route of opium use**			
• Never used opium<br>• Only smoking<br>• Only ingestion<br>• Both smoking and ingestion	1<br>1.37 (0.94 – 2.00)<br>1.05 (0.63 – 1.76)<br>2.00 (0.95 – 4.20)	1<br>1.38 (0.77 – 2.46)<br>1.47 (0.73 – 2.96)<br>1.04 (0.14 – 7.69)	
**Gastric cancer**	**n = 225**	**n = 83**	
**Ever use of opium**			0.590
• Never used opium<br>• Ever use of opium	1<br>1.43 (1.05 – 1.93)	1<br>1.08 (0.51 – 2.24)	
**Route of opium use**			
• Never used opium<br>• Only smoking<br>• Only ingestion<br>• Both smoking and ingestion	1<br>1.41 (1.00 – 1.99)<br>1.45 (0.93 – 2.26)<br>1.46 (0.63 – 3.38)	1<br>1.55 (0.69 – 3.48)<br>0.58 (0.13 – 2.44)<br>-	
**Lung cancer**	**n = 89**	**n = 27**	
**Ever use of opium**			0.464
• Never used opium<br>• Ever use of opium	1<br>2.37 (1.45 – 3.72)	1<br>1.60 (0.48 – 5.38)	
**Route of opium use**			
• Never used opium<br>• Only smoking<br>• Only ingestion<br>• Both smoking and ingestion	1<br>2.12 (1.26 – 3.59)<br>2.49 (1.33 – 4.67)<br>3.52 (1.46 – 8.46)	1<br>0.58 (0.07 – 4.77)<br>4.03 (1.08 – 14.95)<br>-	
**Colon cancer**	**n = 49**	**n = 46**	
**Ever use of opium**			0.500
• Never used opium<br>• Ever use of opium	1<br>0.75 (0.36 – 1.56)	1<br>1.30 (0.43 – 3.88)	
**Route of opium use**			
• Never used opium<br>• Only smoking<br>• Only ingestion<br>• Both smoking and ingestion	1<br>0.89 (0.41 – 1.95)<br>0.53 (0.12 – 2.32)<br>-	1<br>0.50 (0.06 – 3.81)<br>3.16 (0.92 – 10.84)<br>-	
**Brain cancer**	**n = 27**	**n = 53**	
**Ever use of opium**			0.734

• Never used opium<br>• Ever use of opium	1<br>1.10 (0.46 – 2.62)	1<br>1.09 (0.45 – 2.65)	
**Route of opium use**			
• Never used opium<br>• Only smoking<br>• Only ingestion<br>• Both smoking and ingestion	1<br>0.68 (0.21 – 2.14)<br>2.50 (0.88 – 7.02)<br>-	1<br>0.73 (0.21 – 2.49)<br>1.62 (0.47 – 5.55)<br>2.76 (0.34 – 21.95)	
**Pancreatic cancer**	**n = 40**	**n = 38**	
**Ever use of opium**			0.857
• Never used opium<br>• Ever use of opium	1<br>1.85 (0.91 – 3.72)	1<br>1.19 (0.42 – 3.33)	
**Route of opium use**			
• Never used opium<br>• Only smoking<br>• Only ingestion<br>• Both smoking and ingestion	1<br>1.88 (0.86 – 4.09)<br>1.49 (0.48 – 4.63)<br>3.01(0.66 – 13.54)	1<br>1.23 (0.35 – 4.30)<br>1.27 (0.28 – 5.71)<br>-	
**Liver cancer**	**n = 48**	**n = 25**	
**Ever use of opium**			0.205
• Never used opium<br>• Ever use of opium	1<br>1.54 (0.81 – 2.93)	1<br>0.29 (0.03 – 2.34)	
**Route of opium use**			
• Never used opium<br>• Only smoking<br>• Only ingestion<br>• Both smoking and ingestion	1<br>1.00 (0.44 – 2.29)<br>3.36 (1.54 – 7.33)<br>-	1<br>-<br>0.85 (0.10 – 6.76)<br>-	
**Bladder cancer**	**n = 36**	**n = 11**	
**Ever use of opium**			0.715
• Never used opium<br>• Ever use of opium	1<br>2.57 (1.23 – 5.37)	1<br>4.10 (1.03 – 16.22)	
**Route of opium use**			
• Never used opium<br>• Only smoking<br>• Only ingestion<br>• Both smoking and ingestion	1<br>2.31 (1.01 – 5.29)<br>3.36 (1.32 – 8.57)<br>1.62 (0.20 – 12.99)	1<br>4.11 (0.84 – 20.11)<br>4.69 (0.56 – 39.37)<br>-	
**Laryngeal cancer**	**n = 33**	**n = 5**	
**Ever use of opium**			0.437
• Never used opium<br>• Ever use of opium	1<br>2.24 (1.03 – 4.86)	1<br>6.09 (0.67 – 54.82)	
**Route of opium use**			
• Never used opium<br>• Only smoking<br>• Only ingestion<br>• Both smoking and ingestion	1<br>2.30 (0.99 – 5.32)<br>2.07 (0.72 – 5.89)<br>2.40 (0.49 – 11.62)	1<br>4.89 (0.36 – 66.20)<br>9.32 (0.67 – 128.37)<br>-	

Table footnotes:

HR: Hazards Ratio, CI: Confidence Interval, N: Number

‡: this model used age as the time-scale and was adjusted for sex, ethnicity (Turkman/non-Turkman), residence (urban/rural), wealth score quartiles, smoking cigarettes (ever/never), cumulative pack-years of smoked cigarettes (continuous variable), and regular alcohol drinking (never/ever)

Supplementary Table 9. Sensitivity analysis on using opium and risk of different cancer types after dropping the first two years of follow-up in the Golestan Cohort Study			
**Cancer Type**	**Case group n (%)**	**Non-case group n (%)**	**Adjusted ‡ HR (95% CI)**
**All cancers combined (n=1,621)**			
**Ever use of opium**			
• Never used opium	1,211 (74.7)	39,711 (83.6)	1
• Ever use of opium	410 (25.2)	7,752 (16.3)	1.35 (1.19 – 1.54)
**Route of opium use**			
• Never used opium	1,211 (74.7)	39,711 (83.6)	1
• Only smoking	248 (15.3)	5,384 (11.3)	1.31 (1.13 – 1.52)
• Only ingestion	130 (8.0)	1,909 (4.0)	1.41 (1.16 – 1.71)
• Both smoking and ingestion	32 (1.9)	459 (0.9)	1.53 (1.07 – 2.20)
**Gastrointestinal cancers combined (n=796)**			
**Ever use of opium**			
• Never used opium	589 (73.9)	40,333 (83.5)	1
• Ever use of opium	207 (26.0)	7,955 (16.4)	1.36 (1.14 – 1.62)
**Route of opium use**			
• Never used opium	589 (73.9)	40,333 (83.5)	1
• Only smoking	125 (15.7)	5,507 (11.4)	1.35 (1.09 – 1.66)
• Only ingestion	66 (8.2)	1,973 (4.0)	1.34 (1.02 – 1.76)
• Both smoking and ingestion	16 (2.0)	475 (0.9)	1.54 (0.93 – 2.57)
**Respiratory cancers combined (n=133)**			
**Ever use of opium**			
• Never used opium	67 (50.3)	40,855 (83.4)	1
• Ever use of opium	66 (49.6)	8,096 (16.5)	2.07 (1.39 – 3.08)
**Route of opium use**			
• Never used opium	67 (50.3)	40,855 (83.4)	1
• Only smoking	39 (29.3)	5,593 (11.4)	1.99 (1.28 – 3.10)
• Only ingestion	21 (15.7)	2,018 (4.1)	2.20 (1.28 – 3.78)
• Both smoking and ingestion	6 (4.5)	485 (0.9)	2.26 (0.93 – 5.47)
**Esophageal cancer (n=276)**			
**Ever use of opium**			
• Never used opium	199 (72.1)	40,723 (83.4)	1
• Ever use of opium	77 (27.9)	8,085 (16.5)	1.52 (1.13 – 2.04)
**Route of opium use**			
• Never used opium	199 (72.1)	40,723 (83.4)	1
• Only smoking	46 (16.6)	5,586 (11.4)	1.54 (1.09 – 2.18)
• Only ingestion	22 (7.9)	2,017 (4.1)	1.27 (0.80 – 2.03)
• Both smoking and ingestion	9 (3.2)	482 (0.9)	2.65 (1.33 – 5.30)
**Gastric cancer (n=284)**			
**Ever use of opium**			
• Never used opium	200 (70.4)	40,722 (83.4)	1
• Ever use of opium	84 (29.5)	8,078 (16.5)	1.44 (1.08 – 1.91)
**Route of opium use**			
• Never used opium	200 (70.4)	40,722 (83.4)	1
• Only smoking	54 (19.0)	5,578 (11.4)	1.53 (1.11 – 2.12)
• Only ingestion	26 (9.1)	2,013 (4.1)	1.34 (0.87 – 2.07)
• Both smoking and ingestion	4 (1.4)	487 (1.0)	0.96 (0.35 – 2.63)
**Lung cancer (n=96)**			
**Ever use of opium**			
• Never used opium	52 (54.1)	40,870 (83.4)	1
• Ever use of opium	44 (45.8)	8,118 (16.5)	1.96 (1.22 – 3.14)
**Route of opium use**			
• Never used opium	52 (54.1)	40,870 (83.4)	1
• Only smoking	25 (26.0)	5,607 (11.4)	1.80 (1.06 – 3.07)
• Only ingestion	15 (15.6)	2,024 (4.1)	2.26 (1.19 – 4.27)
• Both smoking and ingestion	4 (4.1)	487 (0.9)	2.18 (0.74 – 6.39)
**Colon cancer (n=91)**			
**Ever use of opium**			
• Never used opium	77 (84.6)	40,845 (83.3)	1
• Ever use of opium	14 (15.3)	8,148 (16.6)	0.92 (0.49 – 1.73)
**Route of opium use**			
• Never used opium	77 (84.6)	40,845 (83.3)	1
• Only smoking	10 (10.9)	5,622 (11.4)	0.96 (0.47 – 1.96)
• Only ingestion	4 (4.4)	2,035 (4.1)	1.00 (0.35 – 2.87)
• Both smoking and ingestion	0	491 (1.0)	-
**Brain cancer (n=74)**			
**Ever use of opium**			

• Never used opium<br>• Ever use of opium	58 (78.3)<br>16 (21.6)	40,864 (83.3)<br>8,146 (16.6)	1<br>1.11 (0.59 – 2.11)
**Route of opium use**			
• Never used opium<br>• Only smoking<br>• Only ingestion<br>• Both smoking and ingestion	58 (78.3)<br>6 (8.1)<br>9 (12.1)<br>1 (1.3)	40,864 (83.3)<br>5,626 (11.4)<br>2,030 (4.1)<br>490 (1.0)	1<br>0.64 (0.26 – 1.57)<br>2.25 (1.03 – 4.90)<br>1.08 (0.14 – 8.19)
**Pancreatic cancer (n=69)**			
**Ever use of opium**			
• Never used opium<br>• Ever use of opium	54 (78.2)<br>15 (21.7)	40,868 (83.3)<br>8,147 (16.6)	1<br>1.12 (0.59 – 2.14)
**Route of opium use**			
• Never used opium<br>• Only smoking<br>• Only ingestion<br>• Both smoking and ingestion	54 (78.2)<br>10 (14.4)<br>4 (5.8)<br>1 (1.4)	40,868 (83.3)<br>5,622 (11.4)<br>2,035 (4.1)<br>490 (1.0)	1<br>1.24 (0.59 – 2.58)<br>0.91 (0.31 – 2.66)<br>1.05 (0.14 – 7.89)
**Liver cancer (n=61)**			
**Ever use of opium**			
• Never used opium<br>• Ever use of opium	45 (73.7)<br>16 (26.2)	40,877 (83.3)<br>8,146 (16.6)	1<br>1.31 (0.69 – 2.49)
**Route of opium use**			
• Never used opium<br>• Only smoking<br>• Only ingestion<br>• Both smoking and ingestion	45 (73.7)<br>6 (9.8)<br>10 (16.3)<br>0	40,877 (83.3)<br>5,626 (11.4)<br>2,029 (4.1)<br>491 (1.0)	1<br>0.79 (0.32 – 1.94)<br>2.72 (1.27 – 5.82)<br>-
**Bladder cancer (n=43)**			
**Ever use of opium**			
• Never used opium<br>• Ever use of opium	24 (55.8)<br>19 (44.1)	40,898 (83.4)<br>8,143 (16.6)	1<br>2.46 (1.22 – 4.94)
**Route of opium use**			
• Never used opium<br>• Only smoking<br>• Only ingestion<br>• Both smoking and ingestion	24 (55.8)<br>11 (25.5)<br>8 (18.6)<br>0	40,898 (83.4)<br>5,621 (11.4)<br>2,031 (4.1)<br>491 (1.0)	1<br>2.25 (1.02 – 4.95)<br>3.43 (1.40 – 8.37)<br>-
**Laryngeal cancer (n=37)**			
**Ever use of opium**			
• Never used opium<br>• Ever use of opium	15 (40.5)<br>22 (59.4)	40,907 (83.4)<br>8,140 (16.6)	1<br>2.38 (1.12 – 5.03)
**Route of opium use**			
• Never used opium<br>• Only smoking<br>• Only ingestion<br>• Both smoking and ingestion	15 (40.5)<br>14 (37.8)<br>6 (16.2)<br>2 (5.4)	40,907 (83.4)<br>5,618 (11.4)<br>2,033 (4.1)<br>489 (1.0)	1<br>2.50 (1.12 – 5.60)<br>2.10 (0.75 – 5.87)<br>2.48 (0.52 – 11.90)

Table footnotes:

HR: Hazards Ratio, CI: Confidence Interval, N: Number

‡: this model used age as the time-scale and was adjusted for sex, ethnicity (Turkman/non-Turkman), residence (urban/rural), wealth score quartiles, smoking cigarettes (ever/never), cumulative pack-years of smoked cigarettes (continuous variable), and regular alcohol drinking (never/ever)

Supplementary Table 10. Sensitivity analysis on using opium and risk of different cancer types after excluding cancer cases without histologic confirmation in the Golestan Cohort Study

Cancer Type	Case group n (%)	Non-case group n (%)	Adjusted ‡ HR (95% CI)
**All cancers combined (n=1,464 , 79.8% of the total cases)**			
**Ever use of opium**			
• Never used opium	1,101 (75.2)	40,197 (83.3)	1
• Ever use of opium	363 (24.8)	8,004 (16.6)	1.33 (1.16 – 1.52)
**Route of opium use**			
• Never used opium	1,101 (75.2)	40,197 (83.3)	1
• Only smoking	216 (14.7)	5,529 (11.4)	1.26 (1.08 – 1.48)
• Only ingestion	113 (7.7)	1,996 (4.1)	1.38 (1.12 – 1.70)
• Both smoking and ingestion	34 (2.3)	479 (0.9)	1.80 (1.26 – 2.57)
**Gastrointestinal cancers combined (n=761 , 83.2% of the total cases)**			
**Ever use of opium**			
• Never used opium	567 (74.5)	40,731 (83.2)	1
• Ever use of opium	194 (25.4)	8,173 (16.1)	1.28 (1.06 – 1.54)
**Route of opium use**			
• Never used opium	567 (74.5)	40,731 (83.2)	1
• Only smoking	114 (14.9)	5,631 (11.5)	1.23 (0.99 – 1.53)
• Only ingestion	62 (8.1)	2,047 (4.1)	1.28 (0.97 – 1.70)
• Both smoking and ingestion	18 (2.3)	495 (1.0)	1.72 (1.06 – 2.79)
**Respiratory cancers combined (n=106 , 68.8% of the total cases)**			
**Ever use of opium**			
• Never used opium	51 (48.1)	41,247 (83.2)	1
• Ever use of opium	55 (51.8)	8,312 (16.7)	2.16 (1.38 – 3.37)
**Route of opium use**			
• Never used opium	51 (48.1)	41,247 (83.2)	1
• Only smoking	34 (32.0)	5,711 (11.5)	2.17 (1.33 – 3.54)
• Only ingestion	15 (14.1)	2,094 (4.2)	2.03 (1.08 – 3.82)
• Both smoking and ingestion	6 (4.6)	507 (1.0)	2.54 (1.01 – 6.39)
**Esophageal cancer (n=309 , 90.3% of the total cases)**			
**Ever use of opium**			
• Never used opium	224 (72.4)	41,074 (83.2)	1.43 (1.08 – 1.90)
• Ever use of opium	85 (27.5)	8,282 (16.7)	
**Route of opium use**			
• Never used opium	224 (72.4)	41,074 (83.2)	1
• Only smoking	49 (15.8)	5,696 (11.5)	1.39 (1.00 – 1.95)
• Only ingestion	28 (9.0)	2,081 (4.2)	1.40 (0.92 – 2.13)
• Both smoking and ingestion	8 (2.5)	505 (1.0)	1.99 (0.96 – 4.12)
**Gastric cancer (n=243 , 78.9% of the total cases)**			
**Ever use of opium**			
• Never used opium	181 (74.4)	41,117 (83.2)	1
• Ever use of opium	62 (25.5)	8,305 (16.8)	1.17 (0.75 – 1.61)
**Route of opium use**			
• Never used opium	181 (74.4)	41,117 (83.2)	1
• Only smoking	39 (16.0)	5,706 (11.5)	1.23 (0.85 – 1.78)
• Only ingestion	17 (7.0)	2,092 (4.2)	0.98 (0.58 – 1.65)
• Both smoking and ingestion	6 (2.4)	507 (1.0)	1.57 (0.68 – 3.62)
**Lung cancer (n=76 , 65.5% of the total cases)**			
**Ever use of opium**			
• Never used opium	39 (51.3)	41,259 (83.2)	1
• Ever use of opium	37 (48.6)	8,330 (16.8)	1.87 (1.10 – 3.18)
**Route of opium use**			
• Never used opium	39 (51.3)	41,259 (83.2)	1
• Only smoking	24 (31.5)	5,721 (11.5)	2.00 (1.12 – 3.54)
• Only ingestion	9 (11.8)	2,100 (4.2)	1.54 (0.70 – 3.38)
• Both smoking and ingestion	4 (5.2)	509 (1.0)	2.11 (0.69 – 6.48)
**Colon cancer (n=76 , 80.0% of the total cases)**			
**Ever use of opium**			
• Never used opium	67 (88.1)	41,231 (83.1)	1
• Ever use of opium	9 (11.8)	8,358 (16.8)	0.67 (0.31 – 1.43)
**Route of opium use**			
• Never used opium	67 (88.1)	41,231 (83.1)	1
• Only smoking	7 (9.2)	5,738 (11.5)	0.76 (0.33 – 1.75)
• Only ingestion	2 (2.6)	2,107 (4.2)	0.58 (0.13 – 2.47)
• Both smoking and ingestion	0	513 (1.0)	-
**Brain cancer (n=52 , 65% of the total cases)**			
**Ever use of opium**			

• Never used opium<br>• Ever use of opium	44 (84.6)<br>8 (15.3)	41,254 (83.1)<br>8,146 (15.3)	1<br>0.78 (0.33 – 1,83)
**Route of opium use**			
• Never used opium<br>• Only smoking<br>• Only ingestion<br>• Both smoking and ingestion	44 (84.6)<br>4 (7.6)<br>4 (7.6)<br>0	41,254 (83.1)<br>5,741 (11.5)<br>2,105 (4.2)<br>513 (1.0)	1<br>0.60 (0.20 – 1.79)<br>1.37 (0.45 – 4.11)<br>-
**Pancreatic cancer (n=65 , 83.3% of the total cases)**			
**Ever use of opium**			
• Never used opium<br>• Ever use of opium	45 (69.2)<br>20 (30.7)	41,253 (83.1)<br>8,347 (16.8)	1<br>1.64 (0.89 – 3.02)
**Route of opium use**			
• Never used opium<br>• Only smoking<br>• Only ingestion<br>• Both smoking and ingestion	45 (69.2)<br>13 (20.0)<br>5 (7.6)<br>2 (3.0)	41,253 (83.1)<br>5,732 (11.5)<br>2,104 (4.2)<br>511 (1.0)	1<br>1.73 (0.87 – 3.43)<br>1.32 (0.49 – 3.55)<br>2.30 (0.53 – 10.0)
**Liver cancer (n=51 , 69.8% of the total cases)**			
**Ever use of opium**			
• Never used opium<br>• Ever use of opium	36 (70.5)<br>15 (29.4)	41,262 (83.1)<br>8,352 (16.8)	1<br>1.42 (0.72 – 2.82)
**Route of opium use**			
• Never used opium<br>• Only smoking<br>• Only ingestion<br>• Both smoking and ingestion	36 (70.5)<br>6 (11.7)<br>9 (17.6)<br>0	41,262 (83.1)<br>5,739 (11.5)<br>2,100 (4.2)<br>513 (1.0)	1<br>0.88 (0.35 – 2.21)<br>3.08 (1.35 – 6.99)<br>-
**Bladder cancer (n=43 , 91.4% of the total cases)**			
**Ever use of opium**			
• Never used opium<br>• Ever use of opium	23 (53.4)<br>20 (46.5)	41,275 (83.1)<br>8,347 (16.8)	1<br>2.59 (1.29 – 5.18)
**Route of opium use**			
• Never used opium<br>• Only smoking<br>• Only ingestion<br>• Both smoking and ingestion	23 (53.4)<br>11 (25.5)<br>8 (18.6)<br>0	41,275 (83.1)<br>5,734 (11.5)<br>2,101 (4.2)<br>512 (1.0)	1<br>2.25 (1.02 – 4.97)<br>3.56 (1.45 – 8.72)<br>1.73 (0.21 – 13.68)
**Laryngeal cancer (n=30 , 78.9% of the total cases)**			
**Ever use of opium**			
• Never used opium<br>• Ever use of opium	12 (40.0)<br>18 (60.0)	41,286 (83.1)<br>8,349 (16.8)	1<br>3.06 (1.30 – 7.18)
**Route of opium use**			
• Never used opium<br>• Only smoking<br>• Only ingestion<br>• Both smoking and ingestion	12 (40.0)<br>10 (33.3)<br>6 (20.0)<br>2 (6.6)	41,286 (83.1)<br>5,735 (11.5)<br>2,103 (4.2)<br>511 (1.0)	1<br>2.70 (1.05 – 6.92)<br>3.72 (1.25 – 11.09)<br>4.09 (0.80 – 20.90)

Table footnotes:

HR: Hazards Ratio, CI: Confidence Interval, N: Number

‡: this model used age as the time-scale and was adjusted for sex, ethnicity (Turkman/non-Turkman), residence (urban/rural), wealth score quartiles, smoking cigarettes (ever/never), cumulative pack-years of smoked cigarettes (continuous variable), and regular alcohol drinking (never/ever)
